# AQP4-IgG and MOG-IgG Related Optic Neuritis—Prevalence, Optical Coherence Tomography Findings, and Visual Outcomes: A Systematic Review and Meta-Analysis

**DOI:** 10.3389/fneur.2020.540156

**Published:** 2020-10-08

**Authors:** Angeliki G. Filippatou, Loulwah Mukharesh, Shiv Saidha, Peter A. Calabresi, Elias S. Sotirchos

**Affiliations:** Department of Neurology, Johns Hopkins University School of Medicine, Baltimore, MD, United States

**Keywords:** optic neuritis (ON), optical coherence tomography (OCT), neuromyelitis optica (NMO), neuromyelitis optica spectrum disorder (NMOsd), visual acuity, retina, aquaporin-4 (AQP4) IgG, myelin oligodendrocyte glycoprotein (MOG) IgG associated disease

## Abstract

**Background:** Optic neuritis (ON) is a cardinal manifestation of multiple sclerosis (MS), aquaporin-4 (AQP4)-IgG-, and myelin oligodendrocyte glycoprotein (MOG)-IgG-associated disease. However, the prevalence of AQP4-IgG seropositivity and MOG-IgG seropositivity in isolated ON is unclear, and studies comparing visual outcomes and optical coherence tomography (OCT)-derived structural retinal measures between MS-ON, AQP4-ON, and MOG-ON eyes are limited by small sample sizes.

**Objectives:** (1) To assess the prevalence of AQP4-IgG and MOG-IgG seropositivity among patients presenting with isolated ON; (2) to compare visual outcomes and OCT measures between AQP4-ON, MOG-ON, and MS-ON eyes.

**Methods:** In this systematic review and meta-analysis, a total of 65 eligible studies were identified by PubMed search. Statistical analyses were performed with random effects models.

**Results:** In adults with isolated ON, AQP4-IgG seroprevalence was 4% in non-Asian and 27% in Asian populations, whereas MOG-IgG seroprevalence was 8 and 20%, respectively. In children, AQP4-IgG seroprevalence was 0.4% in non-Asian and 15% in Asian populations, whereas MOG-IgG seroprevalence was 47 and 31%, respectively. AQP4-ON eyes had lower peri-papillary retinal nerve fiber layer (pRNFL; −11.7 μm, 95% CI: −15.2 to −8.3 μm) and macular ganglion cell + inner plexiform layer (GCIPL; −9.0 μm, 95% CI: −12.5 to −5.4 μm) thicknesses compared with MS-ON eyes. Similarly, pRNFL (−11.2 μm, 95% CI: −21.5 to −0.9 μm) and GCIPL (−6.1 μm, 95% CI: −10.8 to −1.3 μm) thicknesses were lower in MOG-ON compared to MS-ON eyes, but did not differ between AQP4-ON and MOG-ON eyes (pRNFL: −1.9 μm, 95% CI: −9.1 to 5.4 μm; GCIPL: −2.6 μm, 95% CI: −8.9 to 3.8 μm). Visual outcomes were worse in AQP4-ON compared to both MOG-ON (mean logMAR difference: 0.60, 95% CI: 0.39 to 0.81) and MS-ON eyes (mean logMAR difference: 0.68, 95% CI: 0.40 to 0.96) but were similar in MOG-ON and MS-ON eyes (mean logMAR difference: 0.04, 95% CI: −0.05 to 0.14).

**Conclusions:** AQP4-IgG- and MOG-IgG-associated disease are important diagnostic considerations in adults presenting with isolated ON, especially in Asian populations. Furthermore, MOG-IgG seroprevalence is especially high in pediatric isolated ON, in both non-Asian and Asian populations. Despite a similar severity of GCIPL and pRNFL thinning in AQP4-ON and MOG-ON, AQP4-ON is associated with markedly worse visual outcomes.

## Introduction

Optic neuritis (ON) is a cardinal manifestation of inflammatory conditions of the central nervous system (CNS), including multiple sclerosis (MS), aquaporin-4 (AQP4)-IgG-, and myelin oligodendrocyte glycoprotein (MOG)-IgG-associated disease (1–3). Early recognition of the underlying etiology of ON has important therapeutic implications, given that treatment approaches vary between these conditions, and therapies that are efficacious in MS may exacerbate or be ineffective in AQP4-IgG- or MOG-IgG-associated disease (4, 5). Furthermore, visual prognosis appears to differ between these conditions, with AQP4-IgG-associated ON (AQP4-ON) typically characterized by worse visual outcomes in comparison to MS-associated ON (MS-ON) and MOG-IgG-associated ON (MOG-ON) (6–8). In patients presenting with classic neuromyelitis optica (NMO) or acute disseminated encephalomyelitis (ADEM)-like phenotypes, clinical suspicion for AQP4-IgG- or MOG-IgG-associated disease is high, but diagnosis may be challenging and delayed in limited forms, such as isolated ON. Notably, the reported prevalence of AQP4-IgG and MOG-IgG seropositivity among patients presenting with isolated ON varies significantly between studies, and the available literature suggests that seropositivity for these antibodies is more common in non-white populations with ON (9, 10).

Optic nerve injury results in thinning of the retinal nerve fiber layer (RNFL), which is mainly composed of the unmyelinated axons of the retinal ganglion cells (RGCs), and the ganglion cell layer, which contains the cell bodies of the RGCs (1). Optical coherence tomography (OCT) is an imaging technique that utilizes near-infrared light to obtain high-resolution images of the retina *in vivo* and enables the quantitative evaluation of individual retinal layers, allowing assessment of the integrity of the RGC axons [peri-papillary RNFL thickness (pRNFL)] and RGC cell bodies [composite thickness of the macular ganglion cell + inner plexiform layer (GCIPL)] (11, 12). OCT studies have generally demonstrated increased severity of pRNFL and GCIPL thinning following AQP4-ON or MOG-ON, as compared to MS-ON (8). However, given the rarity of AQP4-IgG- and MOG-IgG-associated disease, OCT studies have examined relatively small numbers of participants, not permitting an in-depth characterization and comparison of the retinal neuro-axonal injury that occurs in these conditions.

The primary objectives of this systematic review and meta-analysis were as follows: (1) To determine the seroprevalence of AQP4-IgG and MOG-IgG among patients presenting with isolated ON, and to explore variation in prevalence by geographical location/ethnicity. (2) To assess pRNFL and GCIPL thicknesses in AQP4-ON and MOG-ON eyes (including comparisons to MS-ON and healthy controls), and to investigate whether distinct patterns of retinal injury are associated with AQP4-ON or MOG-ON. (3) To compare visual outcomes between AQP4-ON, MOG-ON, and MS-ON eyes.

## Methods

The present systematic review and meta-analysis is reported according to the Preferred Reporting Items of Systematic Reviews and Meta-Analyses (PRISMA) statement and the Meta-analysis of Observational Studies in Epidemiology (MOOSE) guidelines (13, 14).

### Search Strategy and Study Selection

The PubMed electronic database was queried using search algorithms (available in detail in Supplementary Table 1) including the following keywords: “mog,” “myelin oligodendrocyte glycoprotein,” “nmo,” “neuromyelitis optica,” “aquaporin 4,” “aqp4,” “aquaporin-4,” “optic neuritis,” “optical coherence tomography,” “retina,” “nerve fiber layer,” “ganglion cell,” “vision,” “visual outcome,” and “disability.” Databases were last accessed on October 29, 2019.

All retrieved studies were imported into the Covidence platform for study eligibility screening and inclusion. The studies were screened independently by two reviewers (AGF and LM), and in cases of disagreement, another reviewer (ESS) was consulted.

For our first study objective (assessing the prevalence of AQP4-IgG and MOG-IgG seropositivity in isolated ON), we identified all studies that reported the frequency of AQP4-IgG and/or MOG-IgG seropositivity in a cohort of patients presenting with an initial episode of isolated (monosymptomatic) unilateral or bilateral ON. Study exclusion criteria included the following: (1) studies that did not report the number of patients with pre-existing diagnoses of MS or neuromyelitis optica spectrum disorder (NMOSD) or with prior episodes of neurological dysfunction, (2) *n* < 10 participants, and (3) unclear criteria for participant inclusion or inclusion only of selected high-risk patient subgroups (e.g., bilateral or recurrent ON, normal brain MRI). As secondary analyses, we also identified studies reporting the prevalence of AQP4-IgG and MOG-IgG seropositivity in patients presenting with recurrent isolated (unilateral or bilateral) ON or bilateral simultaneous/rapidly sequential ON.

For our second study objective (comparison of OCT measures between AQP4-ON, MOG-ON, and MS-ON eyes), we identified studies that reported OCT measures from patients with AQP4-ON and/or MOG-ON and included data permitting at least one of the following comparisons: (1) AQP4-ON vs. healthy control (HC) eyes, (2) MOG-ON vs. HC eyes, (3) AQP4-ON vs. MOG-ON eyes, (4) AQP4-ON vs. MS-ON eyes, and (5) MOG-ON vs. MS-ON eyes. Comparison of MS-ON vs HC eyes was not performed as this was not the focus of our study and this has been reported in a recent large meta-analysis (15).

Similarly, for our third study objective (comparison of visual outcomes in AQP4-ON, MOG-ON, and MS-ON eyes), studies were included that reported visual outcomes in AQP4-ON and/or MOG-ON and included data permitting at least one of the following comparisons: (1) AQP4-ON vs. MOG-ON, (2) AQP4-ON vs. MS-ON, and (3) MOG-ON vs. MS-ON.

For our analyses of OCT and visual outcomes, we only included articles with assessments of ON eyes performed at least 3 months after an episode of acute ON. For studies that collected the data necessary for our analyses but did not report the results in a manner appropriate for our purposes (e.g., not separating eyes by ON history, reporting combined estimates for AQP4-IgG seropositive and seronegative NMOSD patients), corresponding authors were contacted and were asked to provide additional information. If this information was not made available, these studies were excluded. Additional unpublished data from the cohorts included in the manuscripts was occasionally provided, at the discretion of the corresponding authors. For the OCT component, studies were also excluded if they did not utilize spectral-domain OCT.

When two or more similar studies (fulfilling inclusion criteria) were reported from the same institution or author with unclear participant overlap between studies, authors were contacted to provide clarification. When unable to obtain this information, the publication with the highest number of participants was included in the analysis. Case reports, reviews, or studies published in a non-English language were excluded. Reference lists of relevant review articles were also examined to identify studies that may have been missed during the initial database search.

### Data Extraction and Outcomes

Two investigators (AGF and LM) independently conducted the data extraction, and any discrepancies were resolved by consensus.

For assessment of the prevalence of AQP4-IgG and MOG-IgG in isolated ON, we recorded the total number of patients presenting with an isolated ON in each study (excluding patients with a prior neurological history), and the number of patients that tested positive for AQP4-IgG or MOG-IgG.

The main outcome measures for OCT analyses were the thicknesses (μm) of the pRNFL and the macular GCIPL [or macular ganglion cell layer complex (GCC), which additionally includes the macular RNFL] of eyes with a history of ON, and this information was recorded for each group as the mean ± SD. Additional data on quadrantal pRNFL thicknesses were collected, if available. For studies that reported OCT measures as median/interquartile range and the corresponding authors had not provided the mean ± SD, a normal distribution was assumed to calculate the SD. If macular OCT measures were reported as volumes, they were converted to thicknesses according to the formula: Thickness = Volume/Surface Area. For macular measures, the region of interest varied between studies (e.g., perifoveal area of 3 or 6 mm in diameter, including or excluding the foveal subfield); thus, the surface area was calculated separately for each study, depending on the utilized protocol. While not a primary focus of this study, we also recorded (when available) the prevalence of microcystoid macular pathology (MMP; also referred to as microcystic macular edema in the literature) in AQP4-ON and MOG-ON eyes (16–18).

For visual outcomes, the main outcome measures were the logarithm of the minimum angle of resolution (logMAR) in eyes with a history of ON and the percentage of affected eyes with high-contrast visual acuity (VA) worse than 20/200.

For MOG-IgG serostatus, only studies that reported using cell-based assays (CBAs) for testing were included, whereas for AQP4-IgG serostatus, studies utilized a variety of assays, including CBAs, indirect tissue immunofluorescence, enzyme-linked immunosorbent assay (ELISA) or fluorescence-based immunoprecipitation assay (FIPA).

Data were extracted from cross-sectional cohorts and from a single time point from longitudinal studies (typically the baseline assessment).

### Data Synthesis and Statistical Analysis

For all study objectives, studies of pediatric participants were examined separately.

We estimated the pooled AQP4-IgG and MOG-IgG prevalence in isolated ON separately for Asian and non-Asian populations, given the divergence of prevalence between studies in these populations, and evidence supporting higher prevalence of NMOSD in Asian populations (10). Given the relatively low prevalence of these disorders in some of the included studies (estimates close to 0%), we utilized the variance-stabilizing double arcsine transformation method (19).

OCT measures were handled as continuous variables. Results are presented as mean differences between the groups of interest. OCT measures from different spectral-domain OCT devices were analyzed together, similar to a prior large meta-analysis in MS, given that, at a group level, it appears that data are comparable across devices and segmentation algorithms (15, 20). In terms of macular OCT measures, the GCIPL and GCC were analyzed together, given that the GCIPL accounts for the majority of the thickness of the GCC. Additionally, we estimated the pooled prevalence of MMP in AQP4-ON and MOG-ON eyes.

For studies reporting VA measurements in logMAR format, logMAR was handled as a continuous variable and results are presented as mean differences between groups of interest. For studies reporting visual outcomes as percentage of eyes with VA worse than 20/200, we calculated the relative risk of this unfavorable visual outcome (i.e., VA < 20/200). Studies reporting visual outcomes in any other formats were included in the qualitative, but not the quantitative, synthesis.

All analyses were performed with random effects models, since the heterogeneity was expected to be high due to varying OCT devices, differing scan protocols and macular regions of interest, and differences in the demographic and clinical characteristics of the participants across studies. To minimize the impact of the study heterogeneity, we did not compare OCT measures or visual outcomes across studies; rather, we estimated between-group differences in each study and then performed a pooled analysis of these estimated differences. We assessed for heterogeneity between the included studies using the *I*^2^ estimate. *I*^2^ > 75% was considered to indicate significant heterogeneity.

Statistical analyses were performed with Stata version 16 (StataCorp, College Station, TX). For the meta-analysis of prevalence, the Stata package “metaprop” was used (21).

## Results

### Prevalence of AQP4-IgG and MOG-IgG Seropositivity in Monosymptomatic ON

#### Study Selection and Study Characteristics

A PubMed search identified 1,187 records. Of these, 197 articles were selected and assessed for eligibility at the full-text level. After careful evaluation, 21 studies, comprising 1,876 patients, were included that met the inclusion criteria (22–42). The detailed flow chart is presented in Supplementary Figure 1. For our secondary analysis in patients with recurrent ON, six studies, comprising 510 patients, were included that met our inclusion criteria (26, 43–47). There was an insufficient number of studies/participants to analyze the prevalence of AQP4-IgG or MOG-IgG seropositivity among patients presenting with isolated bilateral simultaneous or sequential ON. The included studies are summarized in Table 1.

**Table 1:** Characteristics of studies included in the meta-analysis for our first study objective (assessing the prevalence of AQP4-IgG and MOG-IgG seropositivity in isolated ON).

**Patients with monosymptomatic ON**										
**Study**	**Time period**	**Study setting**	**Adult/pediatric**	**Age**	**Female sex**	**Race**	**AQP4-IgG assay**	**MOG-IgG assay**	**Bilateral ON**	**Important considerations**
Carnero Contentti et al. (39)	2009–2015	Argentina	Adult	Mean (±SD): 31.6 (±11.1) in AQP4-IgG positive 38.4 (±12.9) in AQP4-IgG negative	47% in AQP4-IgG positive 80% in AQP4-IgG negative	–	Tissue-based indirect IF	–	32%	–
Chen et al. (40)	1988–1991	Multicenter–USA	Adult (18–45)	Mean (±SD): 32.8 (±6.9)	76%	85% Caucasian	CBA	CBA	0%	Recruited only patients with unilateral ON
Chen et al. (41)	2015–2016	China	Pediatric	Range: 5–18. Mean (±SD): 11.8 (±3.3) in MOG-ON; 16.9 (±0.8) in AQP4-ON	70%	–	CBA	CBA	63%	–
Cobo-Calvo et al. (22)	2014–2016	France	Mixed adult pediatric	Median (range): 16.8 (1.7–64.9) for MOG-ON	52% in MOG-IgG positive	93% Caucasian in MOG-IgG positive	CBA	CBA	22% in MOG-IgG positive	–
Dale et al. (23)	–	Australia	Pediatric	Median (range): 8 (1.3–15.3)	51%	–	ELISA	CBA	67%	–
Deschamps et al. (42)	2014–2016	France	Mixed adult pediatric	Range: 16–57	75%	–	CBA	CBA	10%	MOG AQP4 only tested if patient did not meet diagnostic criteria for MS
Ducloyer et al. (24)	2017–2018	France	Adult	Mean (±SD): 35.6 (±13.8)	68%	–	–	CBA	15%	–
Hacohen et al. (25)	2009–2011	UK France	Pediatric	Range: 1.3–15.8	57%		–	CBA	–	–
Jarius et al. (26)	–	Multicenter–Europe	Mixed adult pediatric	Median (range): 34 (14–72)	75%	96% Caucasian	FIPA	–	22%	–
Kim et al. (28)	2013–2014	South Korea	Adult	Mean (±SD): 38.7 (±11.5) in AQP4-IgG positive 42.3 (±14.7) in AQP4-IgG negative	67%	Asian	CBA	–	7%	–
Kim et al. (27)	2007–2016	South Korea	Adult	Mean (±SD): 43 (±13)	63%	–	CBA	–	21%	–
Liu et al. (29)	2014–2016	China	Adult	Range: 18–72	80%	·	CBA	CBA	20%	–
Petzold et al. (30)	1995–2007	UK	Mixed adult pediatric	Range: 15–71	67%	–	CBA	CBA	–	–
Rostasy et al. (31)	2004–2010	Germany Austria	Pediatric	Median (range): 13 (2–18)	73%	–	CBA	CBA	8%	–
Soelberg et al. (32)	2014–2016	Denmark	Mixed adult pediatric	Median (range): 38 (16–66)	69%	100% Caucasian	CBA	CBA	8%	–
Song et al. (33)	2016–2017	China	Pediatric	Mean (±SD): 10.6 (±4.4)	56%	–	CBA	CBA	52%	–
Storoni et al. (34)	2009–2010	UK	Adult	–	–	61% Caucasian 14% African 15% Asian 10% Other	FIPA	–	–	–
Waters et al. (35)	2004–2017	Canada	Pediatric	Median (IQR): 10.8 (6.2–13.9)	51%	–	CBA	CBA	–	–
Zhao et al. (36)	2015–2016	China	Adult	Mean (±SD): 31.3 (±5.3) for MOG-ON 40.7 (±15.3) for AQP4-ON 31.3 (±13.2) for other	71%	–	CBA	CBA	25%	–
Zhou et al. (38)	2013–2014	China	Mixed adult pediatric	Range: 13–73	66%	–	CBA	–	26%	–
Zhou et al. (37)	2009–2010	China	Adult	Median (range): 36.8 (18–73)	66%	–	CBA	–	24%	–
**Patients with recurrent isolated ON**										
Benoilid et al. (43)	2010–2011	France	Adult	Mean (±SD): 33.1 (±14.8)	73%	97% Caucasian	CBA	–	33%	–
de Seze et al. (44)	2005–2007	France	Adult	Mean (±SD): 35.4 (±11.9)	92%	–	Tissue-based indirect IF	–	–	–
Jarius et al. (26)	–	Multicenter - Europe	Mixed adult pediatric	Median (range): 34 (14–72)	75%	96% Caucasian	FIPA	–	22%	
Jitprapaikulsan et al. (45)	2010–2017	USA	Mixed adult pediatric	Range: 12–72	72%	83% Caucasian	CBA	CBA	22%	–
Li et al. (46)	2008–2013	China	Adult	Mean (±SD): 39.0 (±15.4)	75%	–	CBA	–	23%	–
Martinez-Hernandez et al. (47)	2005–2014	Spain	Mixed adult pediatric	Median (range): 28 (5–65)	71%	–	CBA	–	45%	Only recruited patients with normal or nonspecific MRI findings

*AQP4, aquaporin 4; MOG, myelin oligodendrocyte glycoprotein; MS, multiple sclerosis; ON, optic neuritis; CBA, cell-based assay; FIPA, Fluorescence based immunoprecipitation assay; IF, immunofluorescence; ELISA, enzyme-linked immunosorbent assay; MRI, magnetic resonance imaging; IQR, interquartile range; SD, standard deviation*.

#### AQP4-IgG Prevalence in Monosymptomatic ON

**Figure 1:**
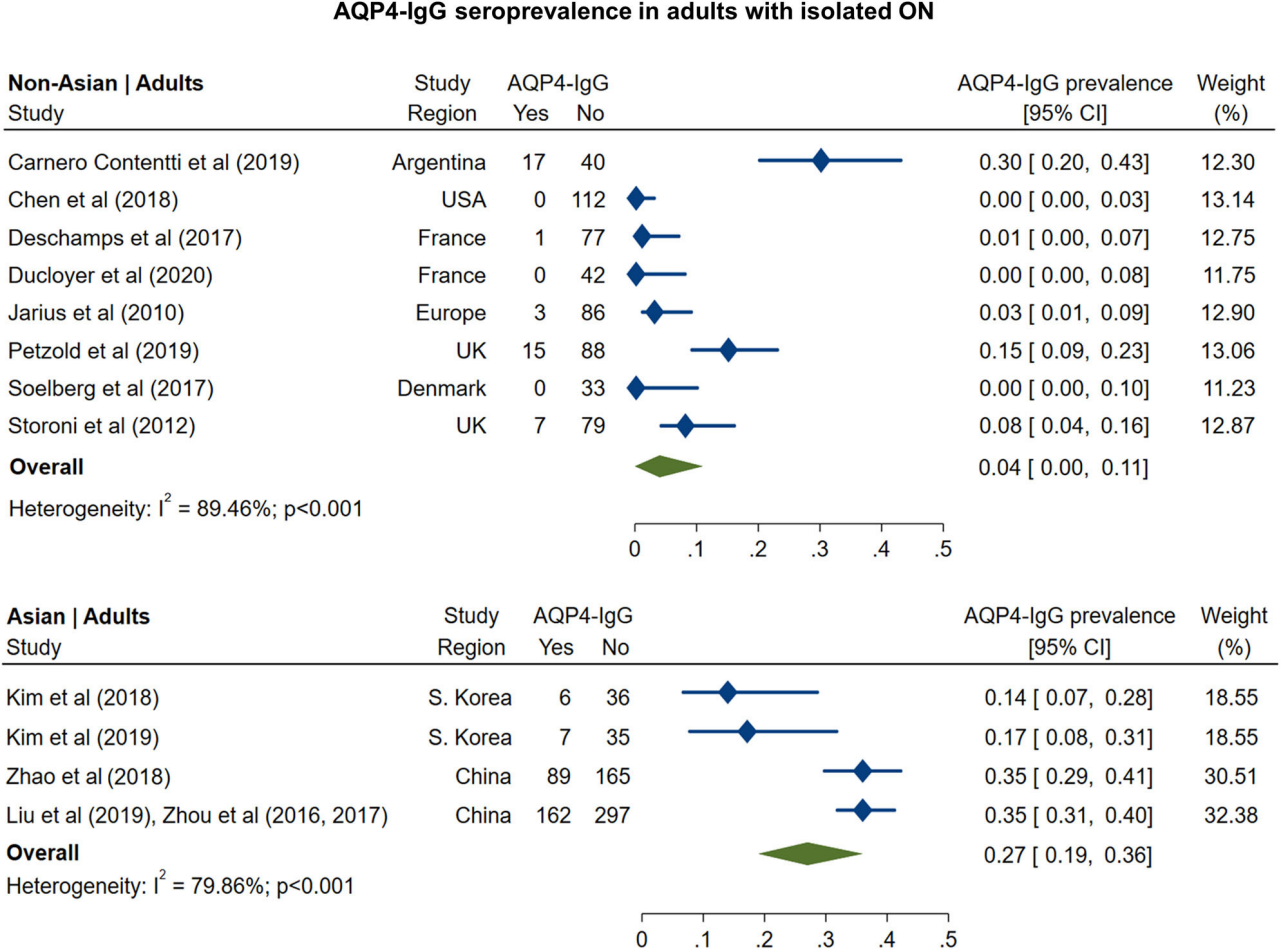
Forest plot of the prevalence of AQP4-IgG seropositivity in adults with monosymptomatic isolated ON.

**Figure 2:**
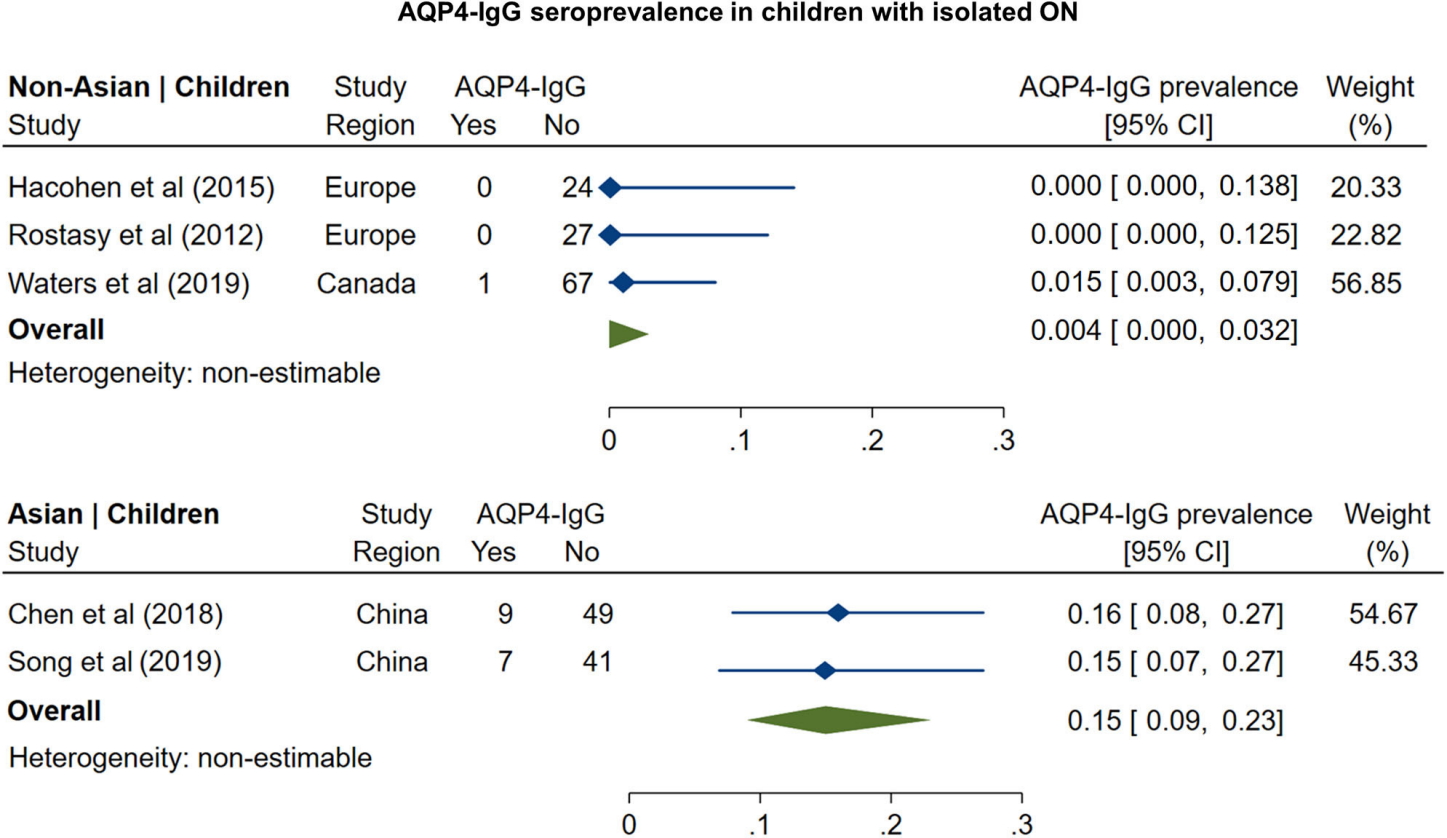
Forest plot of the prevalence of AQP4-IgG seropositivity in children with monosymptomatic isolated ON.

The pooled prevalence of AQP4-IgG seropositivity in adults with isolated ON (Figure 1) was 4% in non-Asian cohorts (95% CI: 0 to 11%) and 27% in Asian cohorts (95% CI: 19 to 36%). In pediatric cohorts (Figure 2), similar to adults, AQP4-IgG seroprevalence was again higher in Asian cohorts (15%; 95% CI: 9 to 23%), whereas in the three available studies of non-Asian populations, the prevalence of AQP4-IgG seropositivity was 0.4% (95% CI: 0 to 3.2%).

#### MOG-IgG Prevalence in Monosymptomatic ON

**Figure 3:**
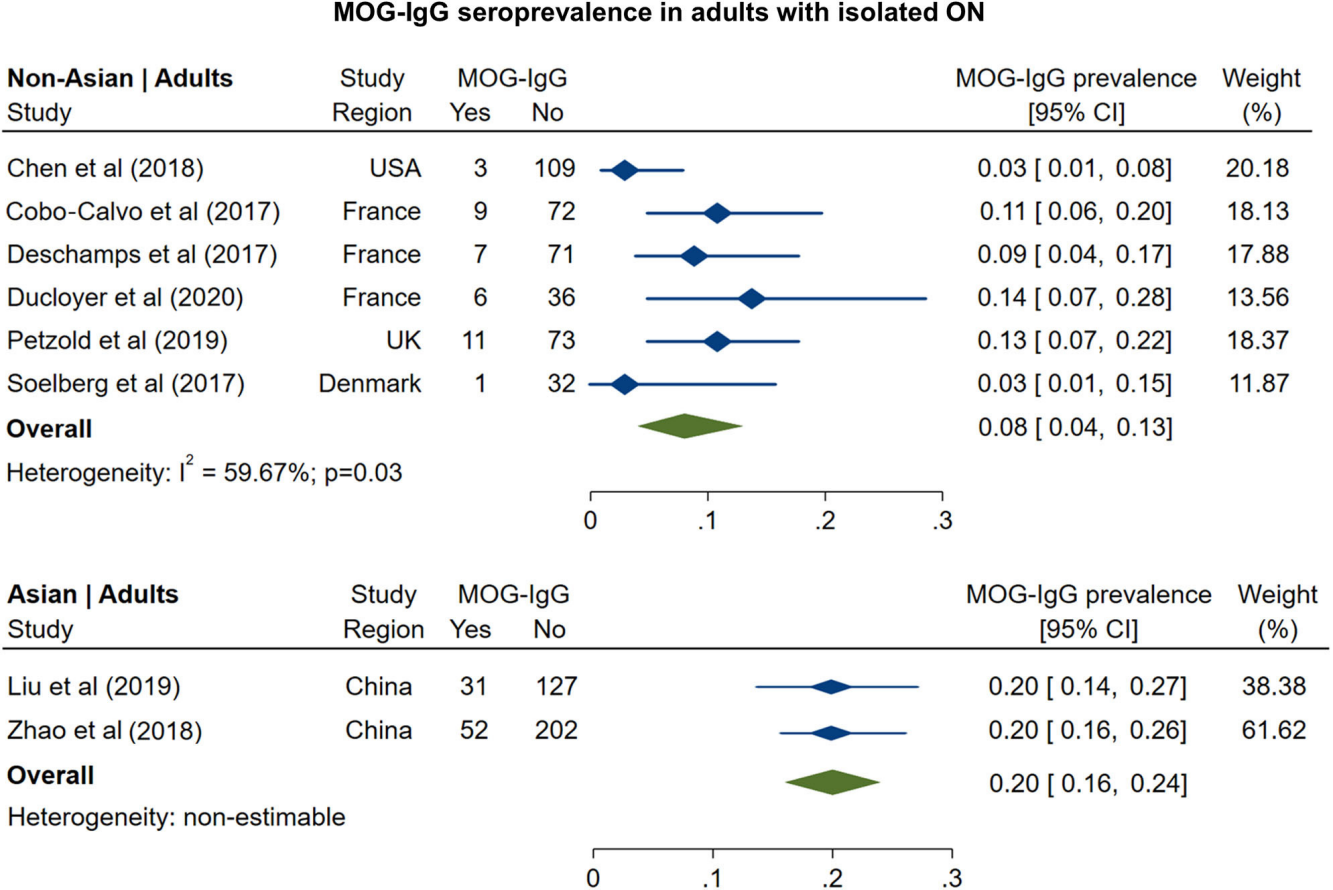
Forest plot of the prevalence of MOG-IgG seropositivity in adults with monosymptomatic isolated ON.

**Figure 4:**
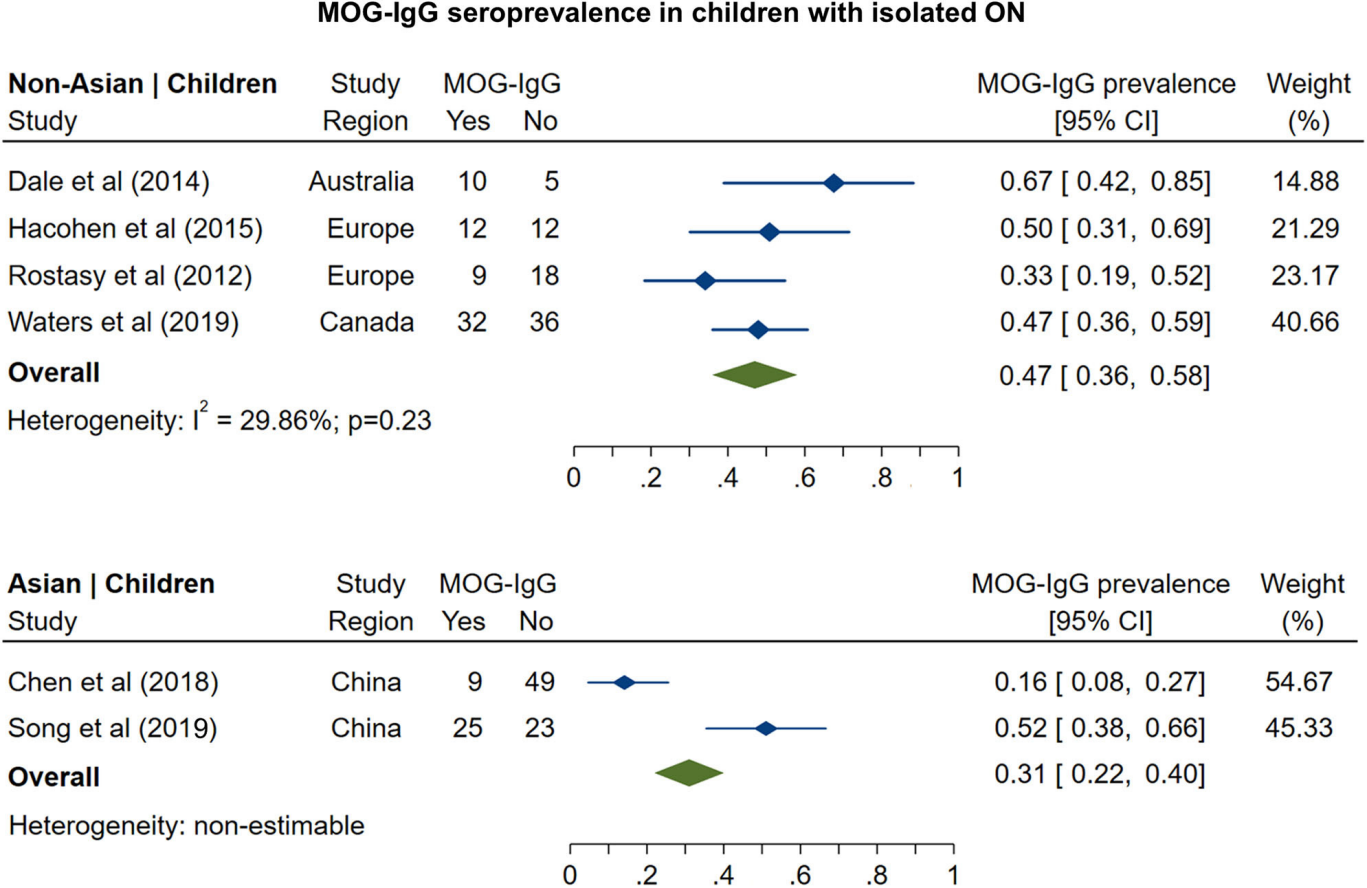
Forest plot of the prevalence of MOG-IgG seropositivity in children with monosymptomatic isolated ON.

The prevalence of MOG-IgG seropositivity in adults with isolated ON (Figure 3) was 8% in non-Asian cohorts (95% CI: 4 to 13%) and 20% in Asian cohorts (95% CI: 16 to 24%). In pediatric cohorts (Figure 4), in contrast to adults, MOG-IgG seroprevalence was higher in non-Asian populations (47%; 95% CI: 36 to 58%) relative to Asian populations (31%; 95% CI: 22 to 40%), but both had higher prevalence compared to adults.

#### AQP4-IgG and MOG-IgG Prevalence in Recurrent Isolated ON

**Figure 5:**
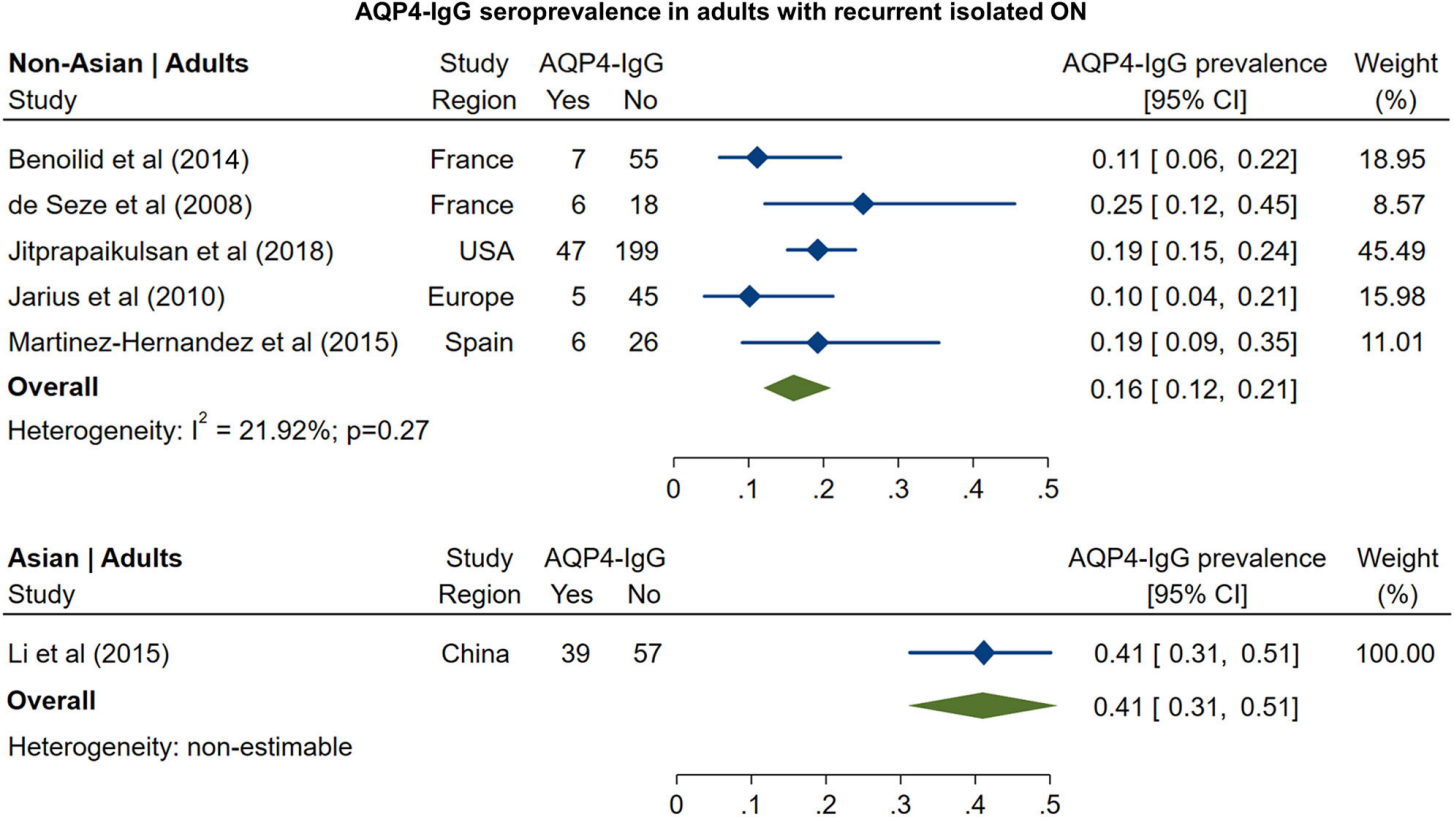
Forest plot of the prevalence of AQP4-IgG seropositivity in adults with recurrent isolated ON.

**Figure 6:**
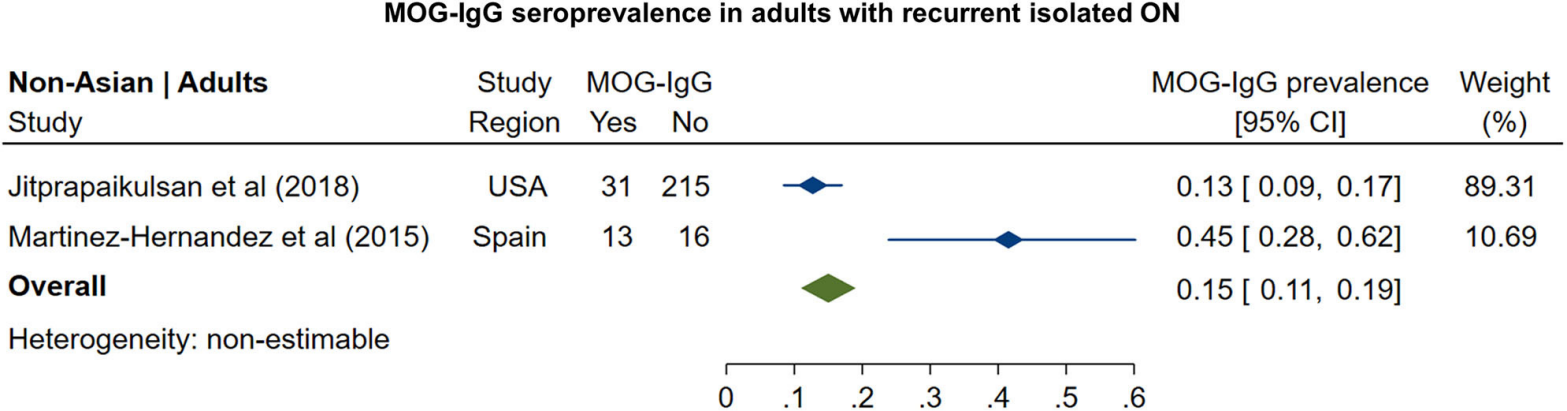
Forest plot of the prevalence of MOG-IgG seropositivity in adults with recurrent isolated ON.

In non-Asian cohorts, the prevalence of AQP4-IgG seropositivity in patients with recurrent isolated ON (Figure 5) was 16% (95% CI: 12 to 21%). Only one study reported the frequency of AQP4-IgG seropositivity in Asian patients with recurrent ON (41%; 95% CI: 31 to 51%). For MOG-IgG, we were able to identify only two studies fulfilling the inclusion criteria; based on these studies (Figure 6), the prevalence of MOG-IgG seropositivity in non-Asian cohorts with recurrent ON was 15% (95% CI: 11 to 19%). No eligible pediatric studies were identified.

### OCT Findings in AQP4-ON and MOG-ON

#### Study Selection and Study Characteristics

A PubMed search identified 351 records. Of these, 98 articles were selected and assessed for eligibility at the full-text level. After careful evaluation, 31 studies were included that met the inclusion criteria (8, 29, 33, 36, 41, 48–73). The detailed flow chart is presented in Supplementary Figure 2. The included studies, comprising a total of 814 HC eyes, 611 AQP4-ON eyes, 237 MOG-ON eyes, and 361 MS-ON eyes, are summarized in Table 2.

**Table 2:** Characteristics of studies included in the meta-analysis for our second study objective (comparison of OCT measures between AQP4-ON, MOG-ON, and MS-ON eyes).

**Study**	**Time period**	**Study setting**	**Adult/** **pediatric**	**Age**	**Female sex**	**Race**	**Device**	**Protocol/ROI**	**MMP**	**Macular** **measure**
Akaishi et al. (48–50)	2005–2013	Japan	Mixed adult pediatric	Mean (±SD): 37.5 (±18.2) in MOG-ON 30 (±9.9) in MS-ON 44.2 (±14.5) in AQP4-ON	75%	–	Topcon (OCT-2000)	–	–	GCC
Chen et al. (41)	2015–2016	China	Pediatric	Range: 5–18	58%	–	Zeiss (Cirrus)	Optic disc cube 200x200 Macular cube 512x128	–	n/a
Deschamps et al. (51)	2011–2016	France	Mixed adult pediatric	Range: 16–63	94% in AQP4-ON 56% in MOG-ON	–	Heidelberg Engineering (Spectralis)	–	–	n/a
Eyre et al. (63)	–	UK Ireland	Pediatric	Median: 8.5 in AQP4-ON MOG-ON 13 in MS-ON	62%	–	Heidelberg Engineering (Spectralis)	–	–	n/a
Havla et al. (52)	2013–2015	Germany France	Adult	Mean (±SD): 41.4 (±14.0) in MOG-ON 39.9± 12.5 in MS-ON 48.3± 8.9 in AQP4-ON 41.5± 13.8 in HC	46% in MOG-ON/MS-ON/HC 79% in AQP4-ON	–	Heidelberg Engineering (Spectralis)	Optic disc: 12° 3.4 mm 50ART Macula: 25 vertical scans ROI: 3 mm ETDRS perifoveal rim	13% of AQP4-ON eyes 46% of MOG-ON eyes 0% of MS-ON eyes	GCIPL
Hokari et al. (53)	2000–2013	Japan	Adult	Median (IQR): 47 (39–62) in AQP4-ON 38 (30–47) in MS-ON	97%	–	Optovue (RTVue-100)	–	–	GCC
Hu et al. (64)	2013–2015	China	Mixed adult pediatric	Mean (±SD): 26.0 (±10.2) in AQP4-ON 28.3 (±3.2) in HC		–	Zeiss (Cirrus)	Optic disc cube 200 x 200 Macular cube 512 x 128	–	GCIPL
Lim et al. (65)	1993–2012	Korea	Adult	Mean (±SD): 30.9 (±11.2) in AQP4-ON 33.7 (±14.8) in MS-ON	73%	–	–	–	–	n/a
Liu et al. (29)	2014–2016	China	Adult	Range: 18–72	80%	–	Zeiss (Cirrus)	–	–	n/a
Martinez-Lapiscina et al. (66)	–	Spain	Adult	Median (IQR): 34.9 [19.4–43.8] in AQP4-ON 54.4 [53.4–58.1] in MOG-ON	66% in AQP4-ON 50% in MOG-ON	-	Heidelberg Engineering (Spectralis)	Optic disc: 12° 100 ART 1536 A scans per B scan). Macula: 20 × 20 degree raster scan 25 horizontal scans (ART?9; 512 A scans per B scan)	0% of AQP4-ON 0% of MOG-ON	GCC
Mekhasingharak et al. (54)	2015–2016	Thailand	Adult	Range: 19–76	92%	–	Zeiss (Cirrus)	Optic disc cube 200x200 Macular cube 512x128	–	GCIPL
Narayan et al. (67)	2009–2018	USA	Pediatric	Mean (±SD): 14.1 (±4.6) in AQP4-ON 18 (±4.9) in MOG-ON	86%	–	Zeiss (Cirrus)	Optic disc cube 200x200 Macular cube 512x128	–	n/a
Oertel et al. (55)	–	Germany UK	Adult	Mean (±SD): 47.3 (±14.4) in AQP4-ON 43.1 (±9.8) in HC	84% in AQP4-ON 79% in HC	76% Caucasian 10% African-Caribbean 8% Asian 2% Middle Eastern 2% mixed 2% unknown	Heidelberg Engineering (Spectralis)	Multiple protocols ROI: 3mm cylinder	–	GCIPL
Oertel et al. (56)	–	Germany France	Mixed adult pediatric	Mean (±SD): 43.1 (±9.8) in HC 40.7 (±13.) in MOG-ON	79% in HC 62.5% in MOG-ON	–	Heidelberg Engineering (Spectralis)	Multiple protocols ROI: 3mm cylinder	30% of MOG-ON eyes	GCIPL
Outteryck et al. (68)	–	France	Adult	Mean (±SD): 44.1 (±9.7) in AQP4-ON 39.7 (±11.3) in MS-ON 38.1 (±12.2) in HC	78% in AQP4-ON 69% in MS-ON 68% in HC	–	Heidelberg Engineering (Spectralis)	ROI: 3mm ETDRS perifoveal rim	15% of AQP4-ON eyes 3% of MS-ON eyes	GCIPL
Pache et al. (57)	–	Germany Denmark	Adult	Mean (±SD): 44.0 (±15.2) in MOG-ON 43.2 (±13.9) in AQP4-ON	97%	100% Caucasian	Heidelberg Engineering (Spectralis)	Optic disc: 12° 768 or 1536 A-scans 16 ≤ ART ≤ 100. Macula: 25° × 30° 61 vertical or horizontal B-scans 768 A-scans per B-scan 9 ≤ ART ≤ 15	19% of AQP4-ON eyes 22% of MOG-ON eyes	GCIPL
Pandit et al. (73)	–	India	Mixed adult pediatric	Median (range): 21 (6–53)	43%	South Asian	Heidelberg Engineering (Spectralis)	Optic disc: 12° 1 536 A-scans ART 100). Macula: 15° x 15° 25 vertical: B-scans ART 100 1024 A-scans per B-scan	21% of MOG-ON eyes	GCC
Peng et al. (58)	–	China	Mixed adult pediatric Excluded patients with AQP4-IgG seropositive NMO (only isolated AQP4-ON)	Range: 17–66	74%	–	Heidelberg Engineering (Spectralis)	ROI: 6 mm ETDRS rim excluding central 1mm	6% of AQP4-ON eyes	GCIPL
Shen et al. (69, 71)	2015–2017	Australia	Adult	Mean (±SD): 48.2 (±16.1) in AQP4-ON/MOG-ON, 43.6 (±10.1) in MS-ON, 39.6 (±14) in HC	68%	–	Heidelberg Engineering (Spectralis)	Optic disc: 3.50 mm Macula: radial star-like scan ROI: Central macular region (2 mm diameter), 6 slices of the star-like scan.	–	GCIPL
Song et al. (33)	2016–2017	China	Pediatric	Mean (±SD): 10.6 (±4.4)	56%	–	Zeiss (Cirrus)	–	–	GCIPL
Sotirchos et al. (8)	2008–2018	USA	Adult	Mean (±SD): 43.7 (±12.7) in AQP4-ON, 43.8 (±13.3) in MOG-ON, 41.5 (±12.6) in MS, 41.5 (±14.1) in HC	78%	61% Caucasian, 34% African American, 5% Other	Zeiss (Cirrus)	Optic disc cube 200 x 200, Macular cube 512 x 128	19% of AQP4-ON eyes, 11% of MOG-ON eyes, 6% of MS-ON eyes	GCIPL
Srikajon et al. (72)	2009–2015	Thailand	Adult	Mean (±SD): 36.7 (±14.0) in AQP4, 34.4 (±13.5) in MS	94%	–	Zeiss (Cirrus)	–	–	n/a
Stiebel-Kalish et al. (59)	2003–2015	Israel	Mixed adult and pediatric	Mean (±SD): 46.3 (±17.6) in AQP4-ON, 41.7 (±9.4) in MOG-ON	69%	–	Zeiss (Cirrus)	Optic disc cube 200x200	–	n/a
Tian et al. (60)	2013–2014	China	Adult ^*^Included only 1 eye per patient	Mean (±SD): 30.5 (±16.7) in MS-ON, 40.5 (±13.6) in AQP4-ON, 32.0 (±13.8) in HC	66%	–	Optovue (RTVue-100)	Optic disc: 4 circular scans (1,024 A-scans/scan), 3.45 mm	–	n/a
vonGlehn et al. (70)	2011–2012	Brazil	Mixed adult and pediatric	Range: 14–76	85%	–	Heidelberg Engineering (Spectralis)	-	–	n/a
Zhang et al. (61)	2012–2017	China	Mixed adult and pediatric	Range: 15–74	74%	-	Zeiss (Cirrus)	Optic disc: 3.45mm	-	n/a
Zhao et al. (36)	2015–2016	China	Mixed adult and pediatric	Mean (±SD):31.3 (±15.3) in MOG-ON,40.7 (±15.3) in AQP4-ON	78%	-	Optovue (RTVue-100)	Optic disc: 3.45mm, 4 circular scans (1024 A-scans/scan)	-	GCIPL
Zhao et al. (62)	2013–2015	China	Adult ^*^Included only eyes with a single ON episode	Median (IQR) 36.5 (21–47) in AQP4–ON	88%	–	Heidelberg Engineering (Spectralis)	Optic disc: 3.5 mm, ≥50 ART. Macula:25 horizontal scans	32% of AQP4-ON eyes	n/a

* *Ganglion Cell Complex (GCC) = macular nerve fiber layer + ganglion cell/inner plexiform layer (GCIPL)*.

*OCT, Optical Coherence Tomography; AQP4, aquaporin 4; MOG, myelin oligodendrocyte glycoprotein; MS, multiple sclerosis; ON, optic neuritis; MMP, microcystoid macular pathology; HC, healthy control; IQR, interquartile range; SD, standard deviation; GCIPL, ganglion cell/inner plexiform layer; GCC, ganglion cell complex; ROI, region of interest; ART, automatic real time; ETDRS, early treatment diabetic retinopathy study*.

#### OCT Measures in Adult ON

As expected, pRNFL and GCIPL thicknesses were lower in AQP4-ON and MOG-ON eyes, as compared with HC eyes (Supplementary Figures 3, 4). The pooled mean pRNFL difference for AQP4-ON eyes was −38.0 μm (95% CI: −46.5 to −29.6 μm) and −35.7 μm (95% CI: −43.1 to −28.4 μm) for MOG-ON eyes. The pooled mean GCIPL difference was −25.8 μm (95% CI: −29.1 to −22.5 μm) for AQP4-ON eyes and −26.7 μm (95% CI: −32.6 to −20.8 μm) for MOG-ON eyes.

**Figure 7:**
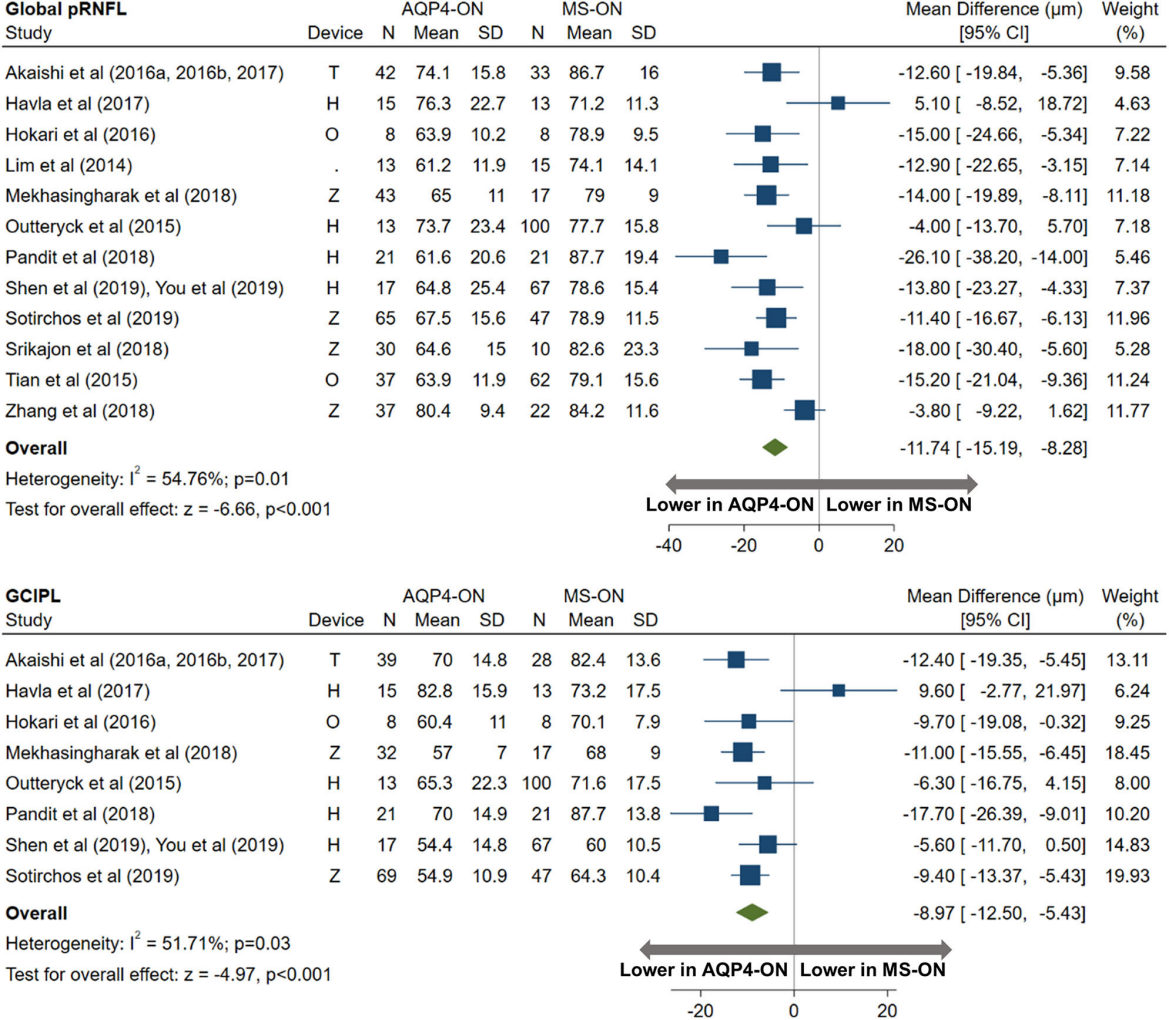
Forest plot of the mean difference in global pRNFL and GCIPL thickness between AQP4-ON and MS-ON. The SD-OCT devices used are indicated as H (Spectralis, Heidelberg Engineering; Heidelberg, Germany), O (RTVue, Optovue Inc; Fremont, CA, USA), and T (3D OCT-2000, Topcon Corporation; Tokyo, Japan), Z (Cirrus, Carl Zeiss Meditec; Dublin, CA, USA).

**Figure 8:**
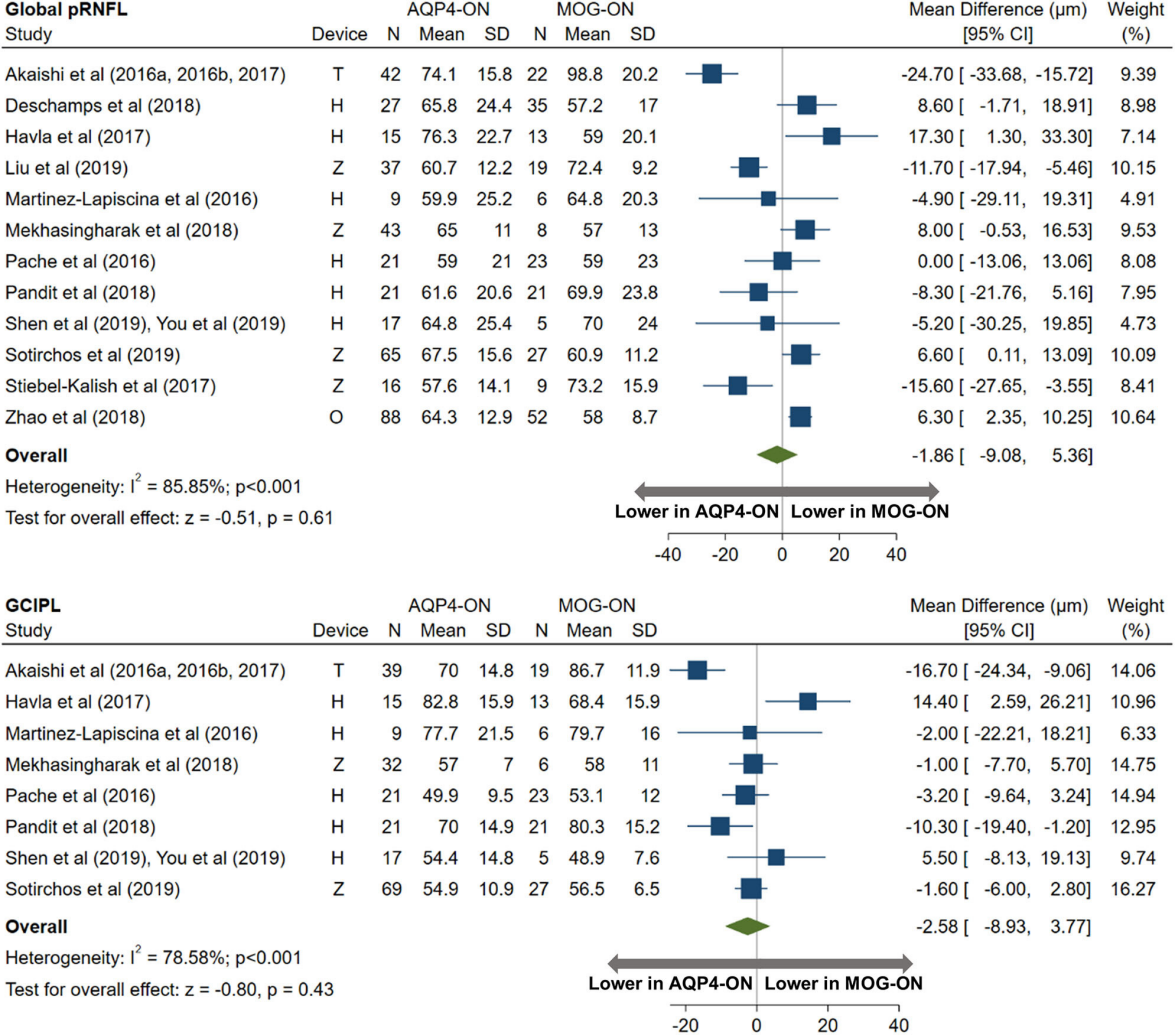
Forest plot of the mean difference in global pRNFL and GCIPL thickness between AQP4-ON and MOG-ON. The SD-OCT devices used are indicated as H (Spectralis, Heidelberg Engineering; Heidelberg, Germany), O (RTVue, Optovue Inc; Fremont, CA, USA), and T (3D OCT-2000, Topcon Corporation; Tokyo, Japan), Z (Cirrus, Carl Zeiss Meditec; Dublin, CA, USA).

**Figure 9:**
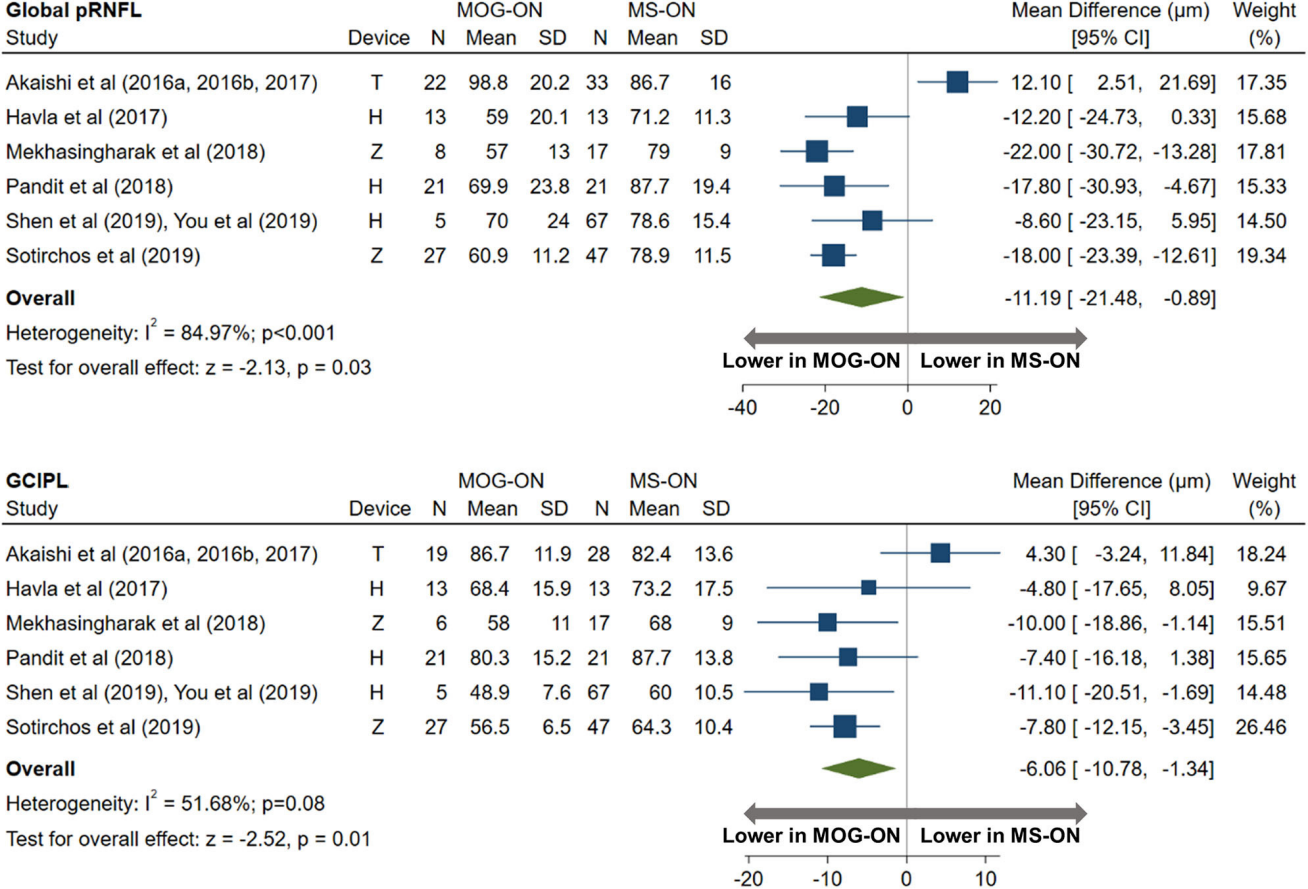
Forest plot of the mean difference in global pRNFL and GCIPL thickness between MOG-ON and MS-ON. The SD-OCT devices used are indicated as H (Spectralis, Heidelberg Engineering; Heidelberg, Germany), O (RTVue, Optovue Inc; Fremont, CA, USA), and T (3D OCT-2000, Topcon Corporation; Tokyo, Japan), Z (Cirrus, Carl Zeiss Meditec; Dublin, CA, USA).

AQP4-ON eyes had lower pRNFL (−11.7 μm; 95% CI: −15.2 to −8.3 μm) and GCIPL (−9.0 μm; 95% CI: −12.5 to −5.4 μm) thicknesses compared with MS-ON (Figure 7), but there were no differences in these OCT measures between AQP4-ON and MOG-ON eyes (pRNFL: −1.9 μm; 95% CI: −9.1 to 5.4 μm; GCIPL: −2.6 μm; 95% CI: −8.9 to 3.8 μm; Figure 8). Similar to AQP4-ON, when comparing MOG-ON to MS-ON eyes (Figure 9), we found that MOG-ON eyes had lower pRNFL (−11.2 μm; 95% CI: −21.5 to −0.9 μm) and GCIPL thicknesses (−6.1 μm; 95% CI −10.8 to −1.3 μm).

When examining quadrantal pRNFL thicknesses, we did not observe any differences between AQP4-ON and MOG-ON (Supplementary Figure 5). However, AQP4-ON was associated with lower nasal, inferior, and superior quadrant pRNFL thicknesses compared with MS-ON (Supplementary Figure 6), but no difference was observed in temporal pRNFL thickness between AQP4-ON and MS-ON eyes (−1.4 μm, 95% CI: −5.9 to 3.1 μm). All quadrantal pRNFL thicknesses were lower in MOG-ON compared to MS-ON eyes (Supplementary Figure 7), but these findings did not achieve statistical significance, likely due to the small sample size.

The prevalence of MMP in ON eyes was reported in a small number of studies. The pooled prevalence of MMP was 15% in AQP4-ON eyes (95% CI: 7 to 24%; *n* = 7 studies) and 21% in MOG-ON eyes (95% CI: 11 to 32%; *n* = 6 studies), which is higher compared to the reported prevalence of MMP in MS-ON eyes (~6%) (16, 17).

#### OCT Measures in Pediatric ON

We were able to identify four studies reporting OCT findings in pediatric ON, and OCT measures could be pooled for three studies (33, 41, 67). Similar to adults, pRNFL thickness did not differ between pediatric AQP4-ON and MOG-ON eyes (7.4 μm, 95% CI: −17.1 to 32.0 μm; Supplementary Figure 8). Further comparisons between groups of interest were not possible based on the available data.

### Visual Outcomes in AQP4-ON and MOG-ON

#### Study Selection and Study Characteristics

A PubMed search identified 624 records. Of these, 202 articles were selected and assessed for eligibility at the full-text level. After careful evaluation, 35 studies were included that met the inclusion criteria (8, 29, 30, 33, 36, 39, 41, 45, 47, 48, 51–54, 57, 59, 63, 65, 66, 68, 69, 71, 72, 74–85). The detailed flow chart is presented in Supplementary Figure 9.

The included studies with their baseline characteristics are summarized in Table 3. In our quantitative synthesis, we included 26 studies comprising 747 AQP4-ON eyes, 426 MOG-ON eyes, and 524 MS-ON eyes.

**Table 3:** Characteristics of studies included in the meta-analysis for our third study objective (comparison of visual outcomes in AQP4-ON, MOG-ON and MS-ON eyes).

**References**	**Time period**	**Study setting**	**Adult/pediatric**	**Age**	**Female sex**	**Race**	**Visual outcome–considerations**
Akaishi et al. (48, 74, 75)	2005–2013	Japan	Mixed adult and pediatric	Mean (±SD): 37.5 (±18.2) in MOG-ON, 30 (±9.9) in MS-ON, 44.2 (±14.5) in AQP4-ON	75%	-	Outcome at eye level
Chen et al. (41)	2015–2016	China	Pediatric	Range: 5–18	58%	-	Outcome at eye level
Cobo-Calvo et al. (76)	2014–2017	France	Adult	Median (range): 36.5 (19–76.8) in MOG-ON, 39.3 (18.2–85) in AQP4-ON	69%	86% Caucasian	Outcome at patient level
Contentti et al. (39)	2009–2015	Argentina	Adult	Mean (±SD): 31.6 (±11.1) in AQP4-ON, 38.4 (±12.9) in other	70%	-	Outcome at patient level
Deschamps et al. (51)	2011–2016	France	Mixed adult and pediatric	Range: 16–63	94% in AQP4-ON, 56% in MOG-ON	-	Outcome at eye level
Eyre et al. (63)	-	UK, Ireland	Pediatric	Median: 8.5 in AQP4-ON and MOG-ON, 13 in MS-ON	62%	-	Outcome at eye level
Falcão-Gonçalves et al. (83)	2004–2016	Brazil	Adult	Median (IQR): 31.6 (22.6–37.4) in AQP4-ON, 27.2 (23.3–37.45) in MS-ON	80%	-	Outcome at eye level
Havla et al. (52)	2013–2015	Germany, France	Adult	Mean (±SD): 41.4 (±14.0) in MOG-ON, 39.9 (±12.5) in MS-ON, 48.3 (±8.9) in AQP4-ON, 41.5 (±13.8) in HC	46% in MOG-ON/ MS-ON/ HC, 79% in AQP4-ON	-	Outcome at eye level
Hokari et al. (53)	2000–2013	Japan	Adult	Median (IQR): 47 (39–62) in AQP4-ON, 38 (30–47) in MS-ON	97%	-	Outcome for number of attacks, not eyes
Ishikawa et al. (77)	2015–2018	Japan	Mixed adult and pediatric	Range: 3–87	84% in AQP4-ON, 51% in MOG-ON	-	Outcome at patient level
Jitprapaikulsan et al. (45)	2000–2017	USA	Mixed adult and pediatric	Range: 5–72	72%	83% Caucasian	Outcome at patient level
Kim et al. (78)	-	South Korea	Adult	Mean (±SD): 39.4 (±12.0) in AQP4-ON, 35.2 (±10.0) in MS-ON	78%	-	Outcome at eye level
Kitley et al. (79)	2010–2013	UK	Adult	Mean (±SD): 32.3 (±17.1) in MOG-ON, 44.9 (±14.8) in AQP4	44% in MOG-ON, 90% in AQP4-ON	66% Caucasian	Outcome at patient level
Lim et al. (65)	1993–2012	Korea	Adult	Mean (±SD): 30.9 (±11.2) in AQP4-ON, 33.7 (±14.8) in MS-ON	73%	-	Outcome at eye level
Liu et al. (29)	2014–2016	China	Adult	Range: 18–72	80%	-	Outcome at eye level
Martinez-Lapiscina et al. (66)	-	Spain	Adult	Median (IQR): 34.9 [19.4–43.8] in AQP4-ON, 54.4 [53.4–58.1] in MOG-ON	66% in AQP4-ON, 50% in MOG-ON	-	Outcome at eye level
Martinez-Hernandez et al. (47)	2005–2014	Spain	Mixed adult and pediatric	Range: 5–65	71%	-	Outcome at patient level
Mekhasingharak et al. (54)	2015–2016	Thailand	Adult	Range: 19–76	92%	-	Outcome at eye level
Merle et al. (84)	-	Martinique	Adult	Mean (±SD): 47.5 (±10.5) in AQP4-ON, 44.5 (±10.1) in MS-ON	87%	-	Outcome at eye level
Outteryck et al. (68)	-	France	Adult	Mean (±SD): 44.1 (±9.7) in AQP4-ON, 39.7 (±11.3) in MS-ON, 38.1 (±12.2) in HC	78% in AQP4-ON, 69% in MS-ON, 68% in HC	-	Outcome at eye level
Pache et al. (57)	-	Germany, Denmark	Adult	Mean (±SD): 44.0 (±15.2) in MOG-ON, 43.2 (±13.9) in AQP4-ON	97%	100% Caucasian	Outcome at eye level
Peng et al. (85)	2014–2015	China	Adult	Mean (±SD): 33 (±12), Range: 30–51	74%	-	Outcome at eye level
Petzold et al. (30)	1995–2007	UK	Mixed adult and pediatric	Range: 15–71	67%	-	Outcome at eye level
Piccolo et al. (80)	2008–2014	UK	Mixed adult and pediatric	Range: 3–59	78%	67% Caucasian	Outcome at patient level
Ramanathan et al. (81)	2001–2014	USA, Australia	Mixed adult and pediatric	Median (range): 15 (3–58)	82%	-	Outcome at patient level
Sepulveda et al. (82)	2013–2015	Spain	Mixed adult and pediatric	Median (range): 39 (10–77)	87%	86% Caucasian	Outcome at patient level
Shen et al. (69) and You et al. (71)	2015–2017	Australia	Adult	Mean (±SD): 48.2 (±16.1) in AQP4-ON/MOG-ON, 43.6 (±10.1) in MS-ON	68%	-	Outcome at eye level
Song et al. (33)	2016–2017	China	Pediatric	Mean (±SD): 10.6 (±4.4)	56%	-	Outcome at eye level
Sotirchos et al. (8)	2008–2018	USA	Adult	Mean (±SD): 43.7 (±12.7) in AQP4-ON, 43.8 (±13.3) in MOG-ON, 41.5 (±12.6) in MS-ON, 41.5 ±14.1 in HC	78%	61% Caucasian, 34% African American, 5% Other	Outcome at eye level
Srikajon et al. (72)	2009–2015	Thailand	Adult	Mean (±SD): 36.7 (±14.0) in AQP4-ON, 34.4 (±13.5) in MS-ON	94%	-	Outcome at eye level
Stiebel-Kalish et al. (59)	2003–2015	Israel	Mixed adult and pediatric	Mean (±SD): 46.3 (±17.6) in AQP4-ON, 41.7 (±9.4) in MOG-ON	69%	-	Outcome at eye level
Zhao et al. (36)	2015–2016	China	Mixed adult and pediatric	Mean (±SD): 31.3 (±15.3) in MOG-ON, 40.7 (±15.3) in AQP4-ON	78%	-	Outcome at eye level

#### Visual Outcomes in Adult ON

**Figure 10:**
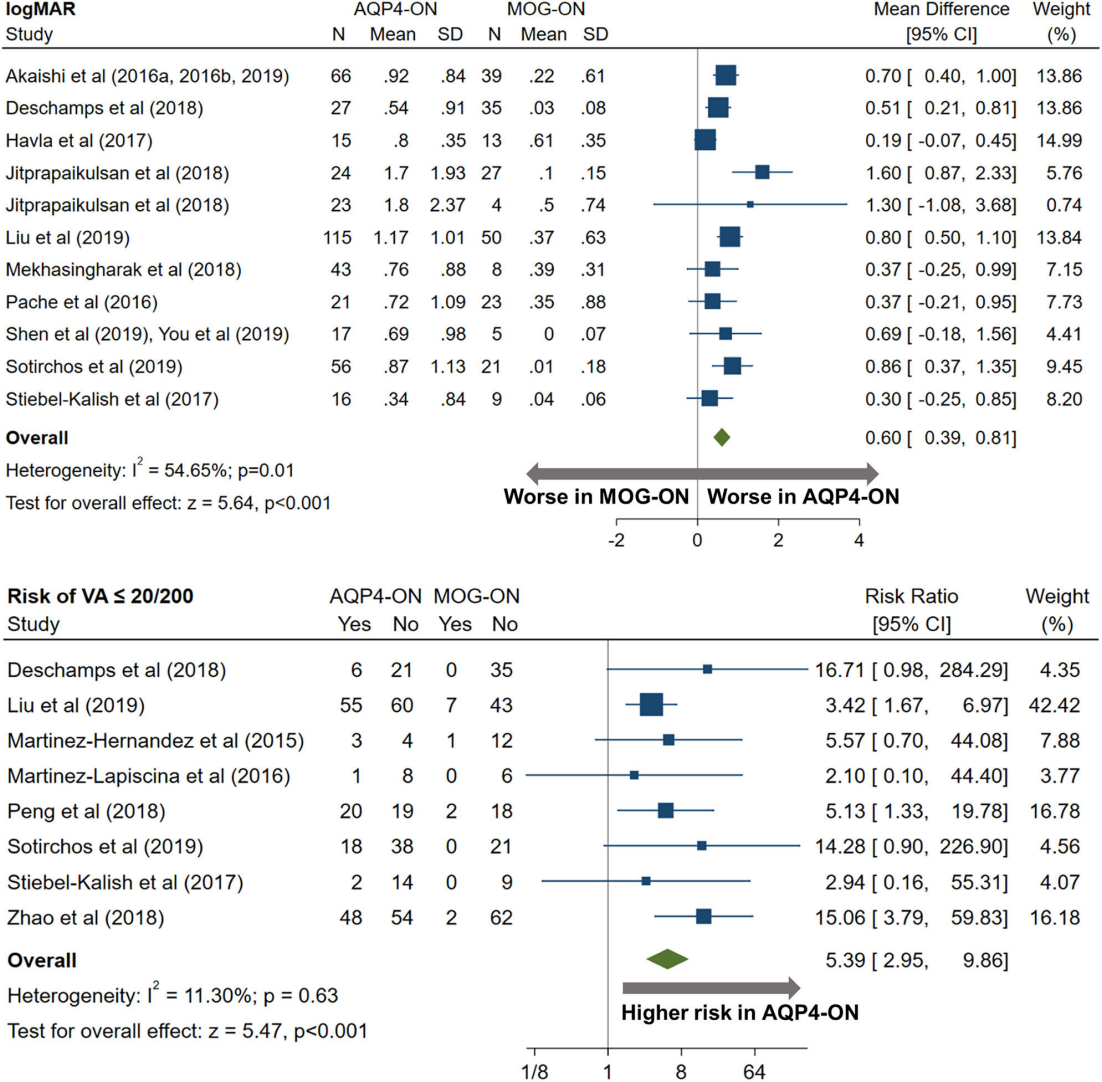
Forest plot of the mean difference in logMAR (high-contrast visual acuity) between AQP4-ON and MOG-ON; forest plot of the relative risk of a poor visual outcome (VA worse than 20/200) in AQP4-ON vs MOG-ON.

**Figure 11:**
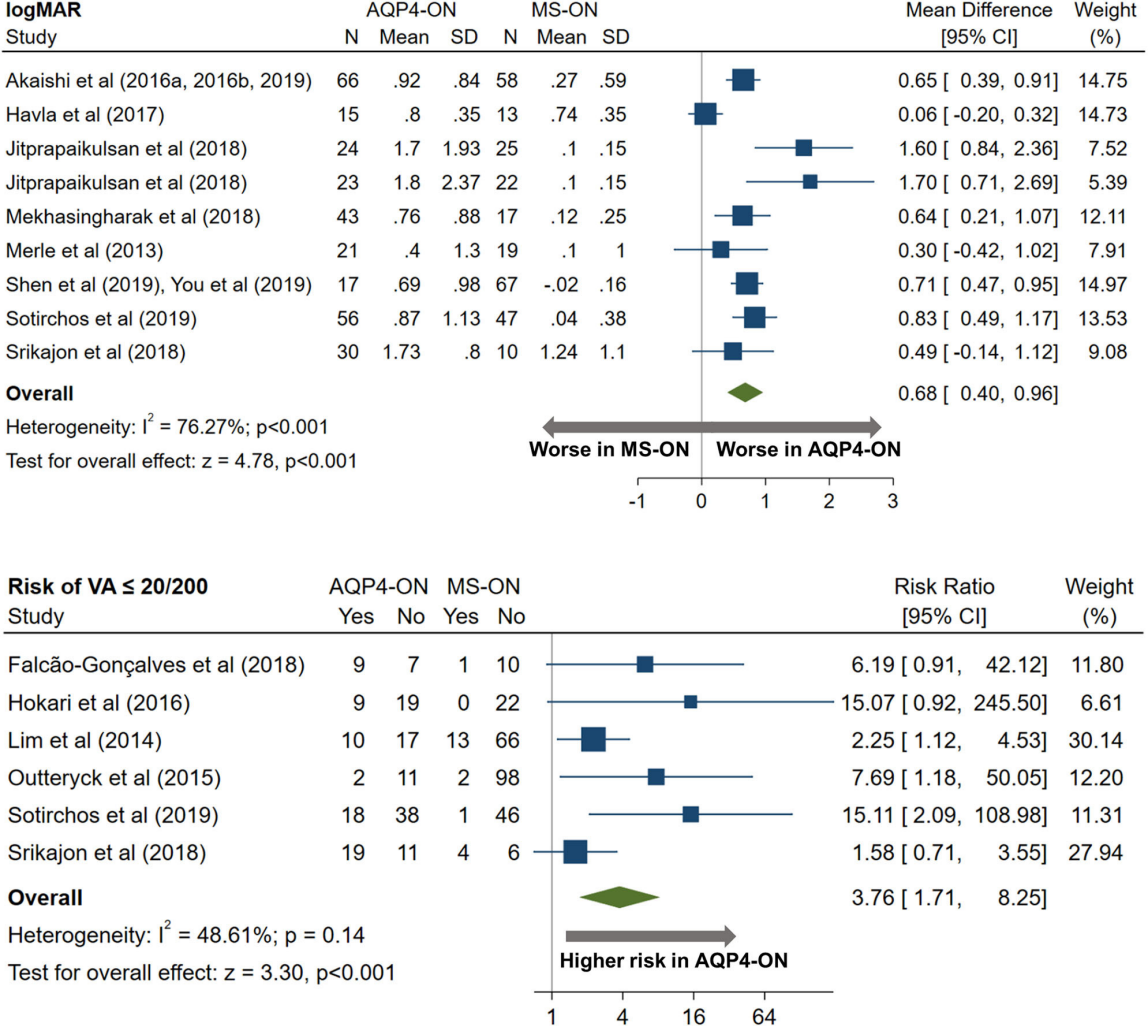
Forest plot of the mean difference in logMAR (high-contrast visual acuity) between AQP4-ON and MS-ON; forest plot of the relative risk of a poor visual outcome (VA worse than 20/200) in AQP4-ON vs MS-ON.

**Figure 12:**
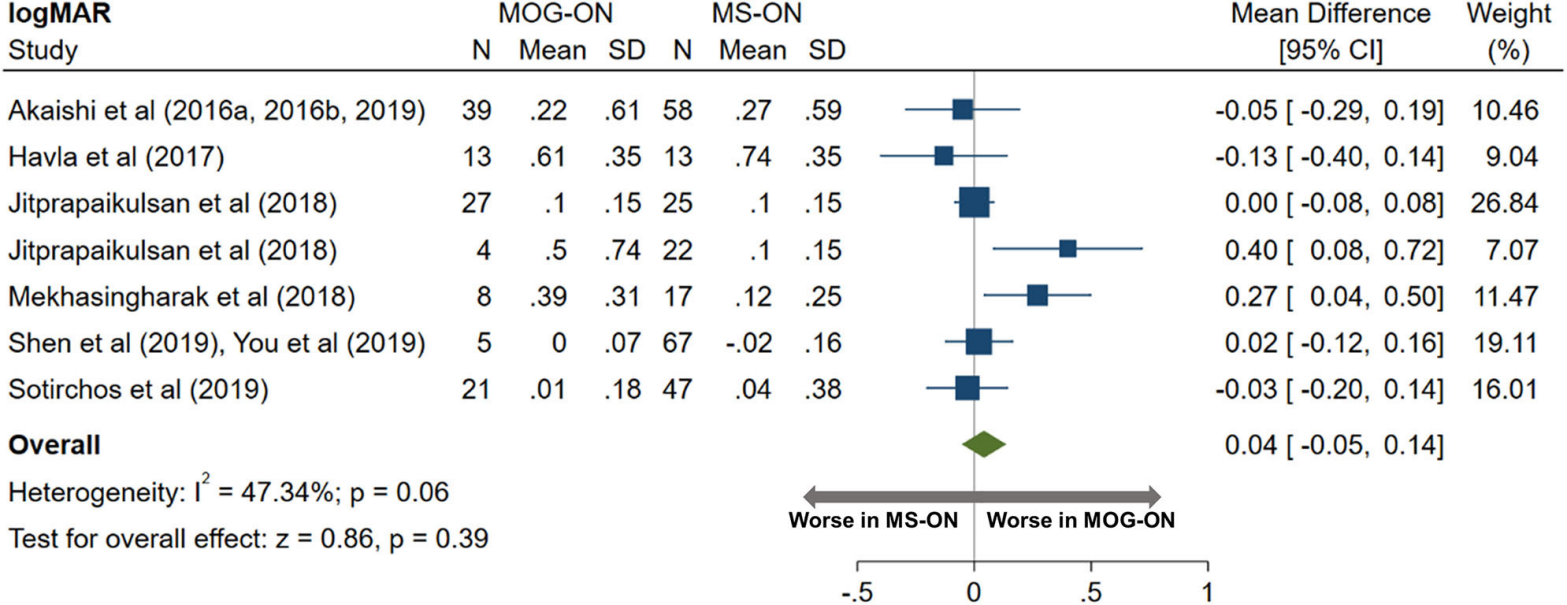
Forest plot of the mean difference in logMAR (high-contrast visual acuity) between MOG-ON and MS-ON.

AQP4-ON eyes had worse high contrast VA when compared to both MOG-ON (mean logMAR difference: 0.60, 95% CI: 0.39 to 0.81) and MS-ON (mean logMAR difference: 0.68, 95% CI: 0.40 to 0.96; Figures 10, 11). Visual outcomes did not differ between MOG-ON and MS-ON (mean logMAR difference: 0.04, 95% CI: −0.05 to 0.14; Figure 12). Moreover, the risk of a poor visual outcome (VA ≤ 20/200) was higher for AQP4-ON compared to MOG-ON [relative risk (RR): 5.39, 95% CI: 2.95 to 9.86; Figure 10] and compared to MS-ON (RR: 3.76, 95% CI: 1.71 to 8.25; Figure 11).

Nine studies were excluded from our quantitative synthesis, since the visual outcomes were not presented in a format that was consistent with the other studies. The findings of the studies are presented in Supplementary Table 2. Importantly, all these studies reported that visual outcomes were markedly better in MOG-ON eyes, as compared with AQP4-ON eyes, in line with the results from the quantitative synthesis.

#### Visual Outcomes in Pediatric ON

We were able to identify three studies reporting visual outcomes in pediatric ON associated with seropositivity for AQP4-IgG and MOG-IgG (33, 41, 63). Similar to adults, the risk of a poor visual outcome (VA ≤ 20/200) was higher for AQP4-ON compared to MOG-ON (RR: 20.11, 95% CI: 4.79 to 84.34), but the sample sizes of the studies were rather small (Supplementary Figure 10).

## Discussion

The present systematic review and meta-analysis revealed variable patterns of seroprevalence of AQP4-IgG and MOG-IgG among patients presenting with isolated ON, with overall higher seroprevalence of both antibodies among Asian populations. Moreover, MOG-IgG-associated ON accounted for a large proportion of pediatric isolated ON (over a third of cases), and high MOG-IgG seroprevalence was noted across the pediatric populations included in our study. Furthermore, despite a similar severity of GCIPL and pRNFL thinning in AQP4-ON and MOG-ON, AQP4-ON was associated with markedly worse visual outcomes, compared to both MOG-ON and MS-ON.

Overall, our results support the idea that AQP4-IgG- and MOG-IgG-associated disorders are not rare entities in Asian populations and are important diagnostic considerations during the initial evaluation of ON in these populations. Notably, cohorts from China comprised the vast majority of the Asian cohorts in our study. However, relatively high seroprevalence of AQP4-IgG and/or MOG-IgG in ON has been reported in several studies (that did not however fully fulfill our inclusion criteria) from Japan, Thailand, Malaysia, and additional Chinese centers (74, 77, 86–89). Importantly, while population-based studies support the notion that Eastern Asian populations have a higher prevalence of NMOSD compared to Caucasian populations, MOG-IgG-associated disease does not appear to exhibit a significant racial preponderance based on data from existing hospital-based studies (90). This suggests that our findings of high AQP4-IgG seroprevalence in ON in Asian populations are likely accounted for by both a higher prevalence of NMOSD and a lower prevalence of MS, whereas for MOG-IgG seroprevalence, the latter may be a more important factor. A noteworthy exception to our finding of overall lower seroprevalence of AQP4-IgG seropositivity in non-Asian populations was the study by Carnero-Contentti et al. (39), which enrolled patients from Buenos Aires, Argentina, and reported an AQP4-IgG seroprevalence of 30% among patients with ON (39). This finding is unexpected, given evidence supporting that the relative frequency of NMO vs. MS in Buenos Aires is low, and similar to that observed in Caucasian populations (91). Notably, this study did not report the ethnic/racial composition of the cohort, and it is possible that referral bias or other factors, which we were unable to detect on our review of the manuscript, contributed to this observation. While the frequency of AQP4-IgG and MOG-IgG seropositivity in ON appears to be lower in non-Asian populations, it remains crucial to consider these entities, especially in patients with atypical characteristics including recurrent or bilateral ON, longitudinally extensive optic nerve lesions, peri-neuritis (MOG-IgG), chiasmal/optic tract involvement (AQP4-IgG >> MOG-IgG), and/or poor visual recovery (AQP4-IgG) (6, 92). As expected, we found markedly higher seroprevalence of AQP4-IgG and MOG-IgG in recurrent isolated ON; however, the number of available studies was small, and mainly limited to non-Asian adult populations. A similar finding was expected in bilateral ON; however, there was an insufficient number of studies/participants eligible to systematically study this. Finally, in children with isolated ON, our results show that MOG-IgG is very commonly detected, across both Asian and non-Asian populations. However, AQP4-IgG seropositivity was exceedingly rare among non-Asian pediatric populations, but relatively common (15%) in Asian pediatric cohorts. The causes of these ethnic and age disparities are poorly understood, but it is likely that there is a genetic component, although environmental factors may also play a role (93, 94).

An important consideration is the fact that the included studies recruited very few patients of African ancestry. This is a critical point since NMOSD occurs frequently in individuals of African ancestry, and African-Americans/Europeans with NMOSD are more likely to experience severe attacks with poor recovery and appear to have higher mortality (95–97). Nevertheless, the frequency of AQP4-IgG and MOG-IgG seropositivity in isolated ON in these populations could not be investigated in the present meta-analysis.

Furthermore, we have found that AQP4-ON and MOG-ON eyes exhibited similarly severely decreased pRNFL and macular GCIPL thicknesses after ON, which was greater than that observed in MS-ON eyes. When examining quadrantal pRNFL thicknesses, we were unable to identify any quadrantal patterns of retinal injury that were specific to MOG-ON. However, when comparing AQP4-ON and MS-ON, AQP4-ON was associated with decreased inferior, superior, and nasal pRNFL thickness, but the temporal pRNFL did not appear to differ between the two groups. This finding suggests that the temporal pRNFL is relatively preserved in AQP4-ON or disproportionally affected in MS-ON. Temporal preponderance of pRNFL damage in MS-ON compared to AQP4-ON was also reported in a study by Schneider et al. (98), which, however, did not fulfill inclusion criteria for our meta-analysis. The pathophysiology underlying the observed differences is not clear; however, the arcuate fibers (located in the superior and inferior quadrants) are commonly injured in vascular optic neuropathies (99). This pattern of quadrantal thinning may suggest that vascular compromise is a mechanism of tissue injury in AQP4-ON. Notably, retinal vascular alterations have been reported *in vivo* in NMO and pathologic studies have identified prominent vascular fibrosis and hyalinization in NMO lesions (99, 100).

Interestingly, and in line with our prior observations (8), we found that, despite a similar severity of pRNFL and GCIPL thinning in AQP4-ON and MOG-ON, visual outcomes clearly diverged between these two entities, with MOG-ON eyes having relatively preserved visual acuity, whereas AQP4-ON eyes experienced markedly worse visual outcomes compared to both MOG-ON and MS-ON. The biological underpinnings of this observation remain unclear. AQP4-IgG-associated disease is recognized as an autoimmune astrocytopathy with secondary demyelination (101). In pathologic studies, a spectrum of changes in astrocytes has been described, including astrocyte necrosis and dystrophic astrocytic profiles (101). AQP4 is highly expressed in the retina, predominantly in retinal astrocytes and Müller glial cells; it is therefore conceivable that AQP4-IgG may cause direct retinal injury. Interestingly, foveal thinning and altered foveal morphology have been reported in AQP4-IgG seropositive eyes without a history of ON, suggesting that subclinical direct retinal involvement may occur in AQP4-IgG-associated disease (102–104). In a pathological study of human retinas, AQP4-IgG seropositivity was associated with loss of AQP4 immunoreactivity on Müller cells, while intravitreal AQP4-IgG injection in mice resulted in reduced AQP4 expression by Müller cells, reactive retinal gliosis and loss of RGCs (53, 105). Notably, AQP4 deletion renders Müller cells incapable of handling osmotic stress and may induce an inflammatory response in the retina (106). These findings suggest that the poor visual prognosis in AQP4-ON may be partially mediated by alterations in the dynamics of astrocyte and Müller cell function. MMP has also been proposed as a factor that is associated with poor outcomes following ON, since MMP eyes have worse visual outcomes and more severe GCIPL and pRNFL thinning (16–18). However, when accounting for GCIPL thickness and ON etiology, MMP does not appear to be independently associated with visual acuity, suggesting that MMP may represent a marker of optic neuropathy severity, rather than a direct contributor to visual dysfunction following ON (8). The prevalence of MMP was reported by a small number of studies included in our meta-analysis but appeared to be overall similar in AQP4-ON (15%) and MOG-ON (21%) and higher in both compared to the reported prevalence in MS-ON (~6%). Further work is needed to clarify the pathoetiology of MMP and whether MMP is causally associated with poor visual outcomes after ON.

Furthermore, we observed an impressive discordance between structural and functional outcomes in MOG-ON; even though MOG-ON was associated with severe pRNFL and GCIPL thinning, high-contrast visual acuity was remarkably preserved and did not differ from MS-ON. Contrary to AQP4, MOG is not expressed in the human retina; therefore, the observed inner retinal thinning is expected to be due to secondary change due to retrograde degeneration and not primary retinal pathology. The pathophysiology underlying the observed structure-function mismatch in MOG-ON is unclear; however, an important consideration is that, with OCT, we are not able to visualize the histological composition of each retinal layer. Therefore, it is conceivable that the relative contributions of the RGCs and their axons to GCIPL and RNFL thicknesses differ between AQP4-ON and MOG-ON, despite a similar severity of retinal layer thinning. In fact, the glial content of the RNFL is considerable and microglia constitute a significant component of the inner plexiform layer, whose thickness is measured as a composite with the ganglion cell layer as GCIPL (107, 108). Given the markedly different pathogenic mechanisms in these disorders, it is conceivable that the observed discrepancies may be related to differences in glial activation and migration, resulting in differing compositions of the pRNFL and the GCIPL and, consequently, different functional capacity of the retina. Another important consideration is that there is a floor effect present for OCT measures, and a single AQP4-ON or MOG-ON attack can lead to marked pRNFL and GCIPL atrophy, while subsequent attacks may not lead to appreciable changes in inner retinal layer thicknesses, despite worsening visual function (109). Analyses comparing visual and structural measures between groups after a single attack of ON would be useful to address this issue; however, the vast majority of studies included in our meta-analysis did not report OCT or visual acuity separately for patients with single and recurrent ON. However, since both AQP4-ON and MOG-ON frequently relapse, we do not expect that this may have significantly affected our findings when comparing outcomes in AQP4-ON vs. MOG-ON, although this may have influenced comparisons with MS-ON (6).

In this meta-analysis, we also attempted to examine OCT findings and visual outcomes in pediatric ON associated with AQP4-IgG and MOG-IgG seropositivity. However, this population has not been studied extensively and a systematic review of the literature revealed only four studies, with small numbers of participants (33, 41, 63, 67). OCT measures could be pooled for three of these, two of which included Asian children (33, 41). Therefore, our meta-analysis is clearly underpowered to study characteristics of pediatric AQP4-ON and MOG-ON. Nevertheless, the OCT findings and visual outcomes appear to be similar to those observed in adults. The inclusion of pediatric cases should be an important consideration for future studies, especially since MOG-IgG antibodies are commonly detected in children with ON.

Despite the strengths of the present report, several limitations must be acknowledged. Firstly, the majority of the included prevalence studies were performed at tertiary academic referral centers, with clinical expertise in neuro-ophthalmology. Therefore, it is conceivable that the patients who were recruited in these studies are not a representative sample of patients presenting with isolated ON and are likely enriched for cases with increased severity or atypical characteristics. Thus, it is possible that our results may overestimate the true prevalence rate of these disorders in the general population due to referral bias. This issue should also be considered when interpreting the OCT and visual outcomes, since patients with more severe attacks of ON and poor recovery are potentially more likely to be referred to a tertiary center for further management, and mild cases with favorable outcomes may be underrepresented in the existing literature. Furthermore, between-study heterogeneity was considerable in almost all pooled analyses of OCT measures or visual outcomes. A potential source of heterogeneity in analyses of OCT measures is the fact that the included studies utilized a variety of spectral-domain OCT devices, as well as scanning and segmentation protocols. Moreover, participants' demographics and clinical characteristics varied considerably between studies and it is likely that there is variability in the phenotype, disease course, and outcomes among different racial or age groups. To minimize the impact of these differences on our results, we did not compare OCT measures or visual outcomes across studies; rather, we estimated the differences in retinal layer thicknesses or logMAR between groups that were included in the same study and performed a pooled analysis of these estimated differences. In analyses of OCT measures and visual outcomes, we were also notably unable to account for the number of ON attacks, since some studies included patients with a single event, while others recruited patients with multiple ON episodes. It is expected that the number of ON episodes has an impact on OCT findings and final visual acuity, especially since recurrent ON in common in cases of AQP4-ON and MOG-ON; this should be a consideration in future studies. Additionally, even though we attempted to analyze findings in adult ON separately from pediatric ON, some studies (noted in Tables 1–3) recruited mixed adult and pediatric or adolescent populations; this is an important consideration when attempting to draw conclusions regarding potential differences in the characteristics of these disease entities between these age groups. Finally, AQP4-IgG serostatus was determined using a variety of assays, including ELISA in some studies, which is known to have an inferior performance in terms of sensitivity and specificity compared to CBAs (110, 111). This is a relevant point, since the use of an assay with sub-optimal diagnostic accuracy may have led to misclassification of patients. Nevertheless, the majority of studies included in our meta-analysis (including 79% of studies assessing the prevalence of AQP4-IgG in ON) utilized CBA to determine the AQP4-IgG serostatus of their participants. MOG-IgG serostatus was determined exclusively using CBAs with full-length human MOG, given that MOG-IgG detected by ELISA or Western blot lacks disease specificity. Notably, commonly used MOG-IgG CBAs demonstrate overall good agreement for high-positive and negative samples, although agreement is lower for borderline results, and this is another factor that could potentially influence diagnostic accuracy in the included studies (112).

## Conclusions

Our systematic review and meta-analysis provides a comprehensive overview of the epidemiology and structural and functional outcomes in ON associated with AQP4-IgG and MOG-IgG seropositivity. Our findings support the idea that AQP4-IgG- and MOG-IgG-related disease are more common causes of ON in Asian vs. non-Asian populations and that MOG-IgG seroprevalence is especially high in pediatric ON, and we provide estimates of seroprevalence in these groups. We have also shown that MOG-ON and AQP4-ON are associated with similar severity of retinal thinning; however, visual outcomes appear to be markedly worse in AQP4-ON. Future studies should seek to investigate the pathoetiology of these findings, as well as to provide insights regarding optimal acute and chronic treatment strategies for these disorders.

## Data Availability Statement

The datasets generated for this study are available on request to the corresponding author.

## Author Contributions

AF and ES: study conception and design, data acquisition, analysis and interpretation, drafting, and revision of the manuscript for important intellectual content. LM: data acquisition and interpretation and revision of the manuscript for important intellectual content. SS and PC: data interpretation and revision of the manuscript for important intellectual content. All authors contributed to the article and approved the submitted version.

## Conflict of Interest

SS had received consulting fees from Medical Logix for the development of CME programs in neurology and had served on scientific advisory boards for Biogen, Genzyme, Genentech Corporation, EMD Serono, and Celgene. SS was the PI of investigator-initiated studies funded by Genentech Corporation and Biogen and received support from the Race to Erase MS foundation. SS had received equity compensation for consulting from JuneBrain LLC, a retinal imaging device developer. SS was also the site investigator of a trial sponsored by MedDay Pharmaceuticals. PC had received consulting fees from Disarm Therapeutics and Biogen and was PI on grants to JHU from Biogen and Annexon. ES had served on scientific advisory boards for Viela Bio and Genentech.The remaining authors declare that the research was conducted in the absence of any commercial or financial relationships that could be construed as a potential conflict of interest. The handling Editor declared a past collaboration with the authors.

## Supplementary Material

Supplementary Figure 1 Study selection for our first study objective (assessing the prevalence of AQP4-IgG and MOG-IgG seropositivity in isolated ON).

Supplementary Figure 2 Study selection for our second study objective (comparison of OCT measures between AQP4-ON, MOG-ON and MS-ON eyes).

Supplementary Figure 3 Forest plot of the mean difference in global pRNFL and GCIPL thickness between AQP4-ON and HC.

Supplementary Figure 4 Forest plot of the mean difference in global pRNFL and GCIPL thickness between MOG-ON and HC.

Supplementary Figure 5 Forest plot of the mean difference in quadrantal pRNFL thicknesses between AQP4-ON and MOG-ON.

Supplementary Figure 6 Forest plot of the mean difference in quadrantal pRNFL thicknesses between AQP4-ON and MS-ON.

Supplementary Figure 7 Forest plot of the mean difference in quadrantal pRNFL thicknesses between MOG-ON and MS-ON.

Supplementary Figure 8 Forest plot of the mean difference in global pRNFL thickness between AQP4-ON and MOG-ON in pediatric ON.

Supplementary Figure 9 Study selection for our third study objective (assessment of the visual outcome in AQP4-ON, MOG-ON and MS-ON eyes).

Supplementary Figure 10 Forest plot of the relative risk of a poor visual outcome (VA worse than 20/200) in AQP4-ON vs MOG-ON in pediatric ON.

Supplementary Table 1 Search terms.

Supplementary Table 2 Visual outcome in AQP4-ON, MOG-ON and MS-ON; qualitative synthesis.

## Abbreviations

AQP4: aquaporin 4
MOG: myelin oligodendrocyte glycoprotein
MS: multiple sclerosis
ON: optic neuritis
pRNFL: peripapillary nerve fiber layer
GCIPL: ganglion cell/inner plexiform layer
VA: visual acuity
OCT: optical coherence tomography
logMAR: logarithm of the minimum angle of resolution
N: number of eyes
SD: standard deviation
CI: confidence interval.
