# Supplementary figures and images for: AQP4-IgG and MOG-IgG Related Optic Neuritis—Prevalence, Optical Coherence Tomography Findings, and Visual Outcomes: A Systematic Review and Meta-Analysis
Source: Front Neurol. 2020 Oct 8;11:540156. doi: 10.3389/fneur.2020.540156 (PMC7578376; doi:10.3389/fneur.2020.540156)

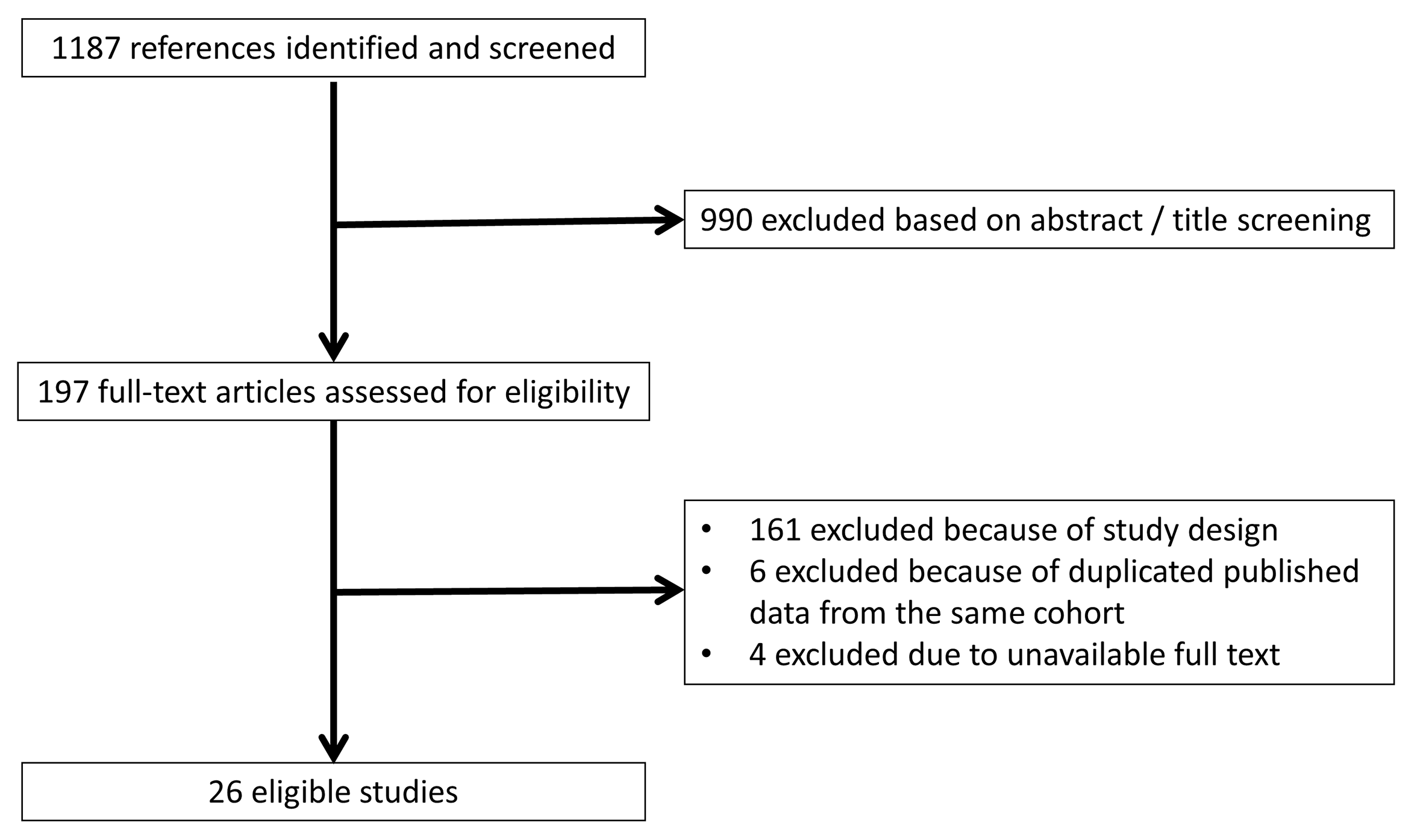

Supplement: Supplementary Figure 1 — Study selection for our first study objective (assessing the prevalence of AQP4-IgG and MOG-IgG seropositivity in isolated ON). [file Image_1.TIF]

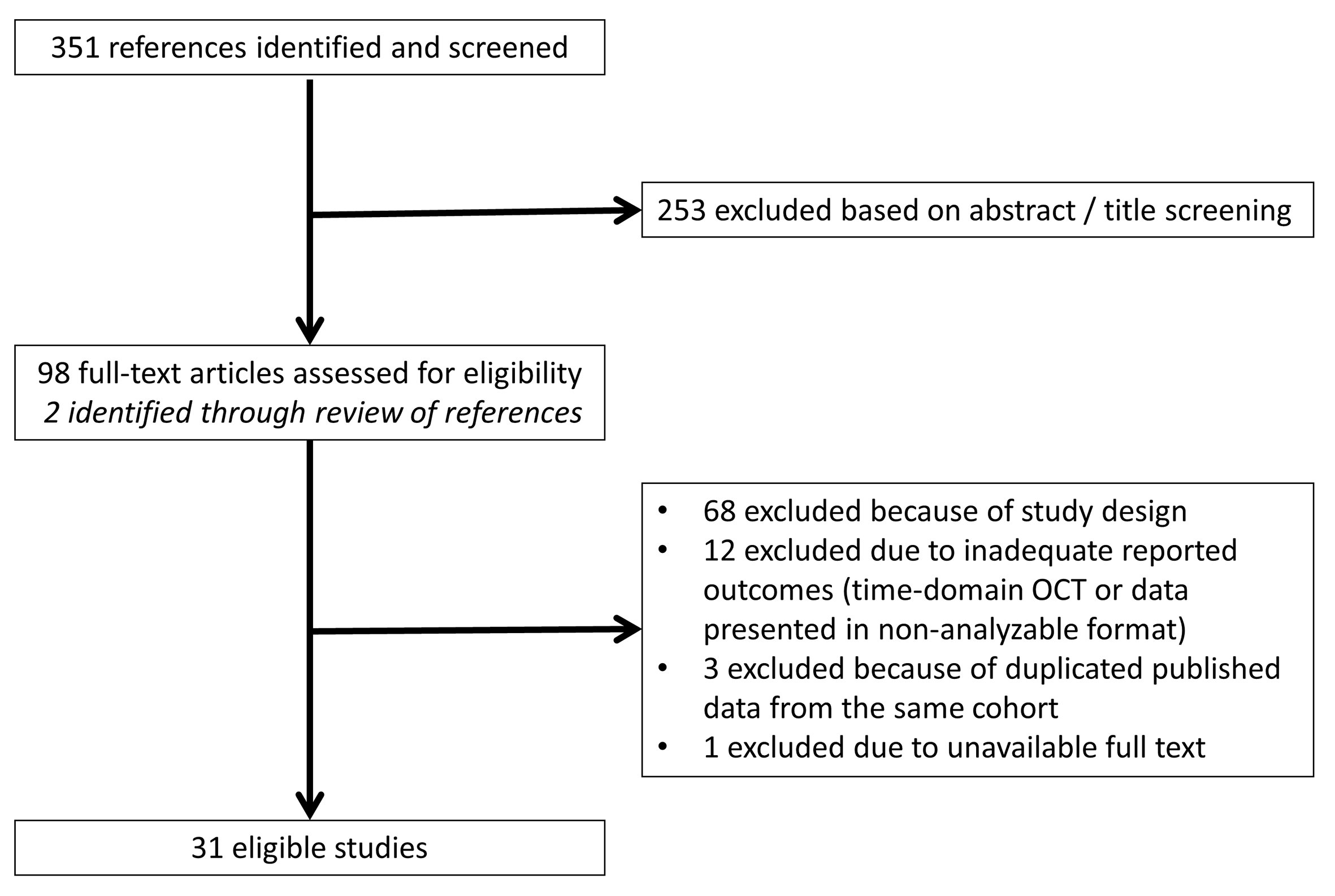

Supplement: Supplementary Figure 2 — Study selection for our second study objective (comparison of OCT measures between AQP4-ON, MOG-ON and MS-ON eyes). [file Image_2.TIF]

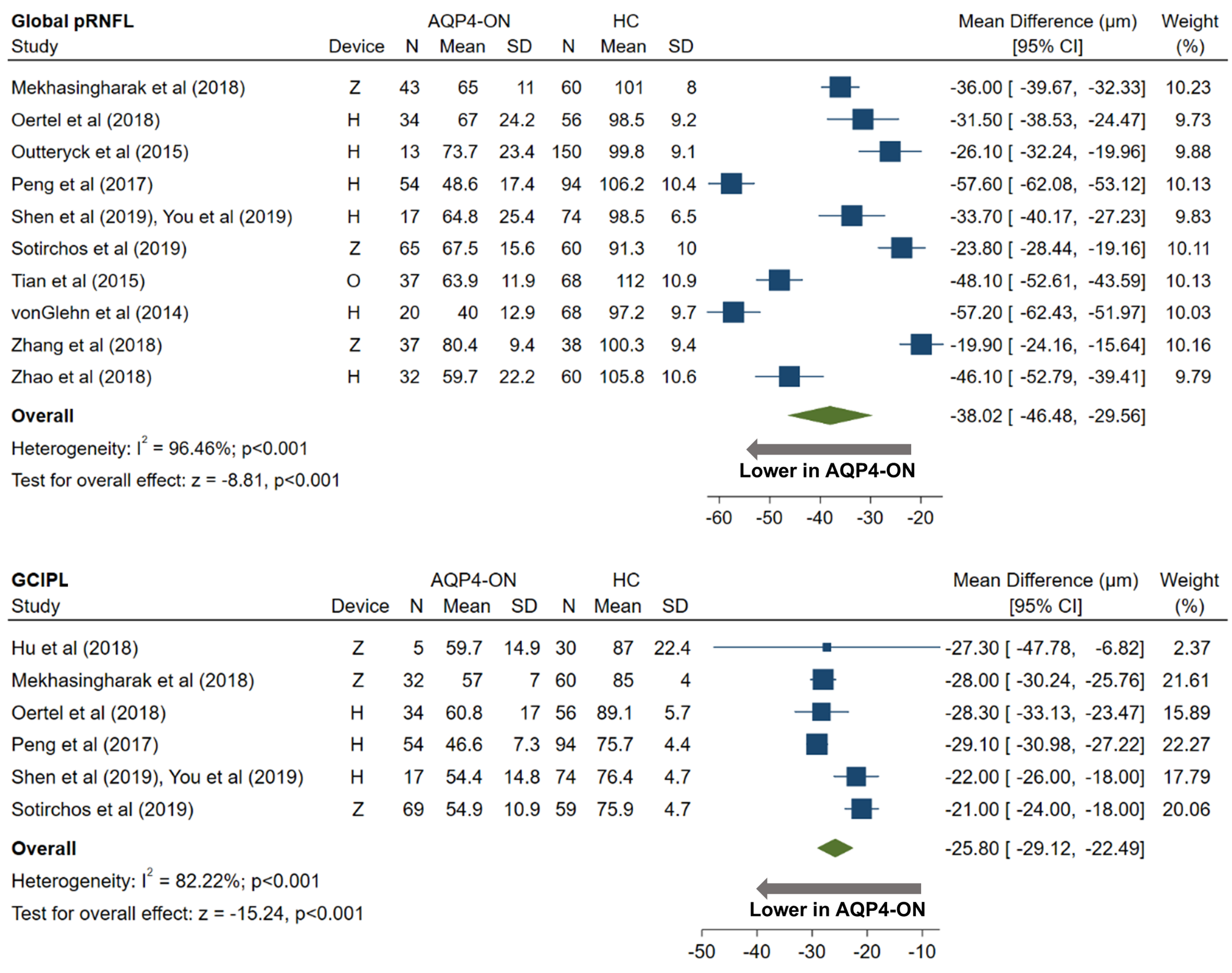

Supplement: Supplementary Figure 3 — Forest plot of the mean difference in global pRNFL and GCIPL thickness between AQP4-ON and HC. [file Image_3.TIF]

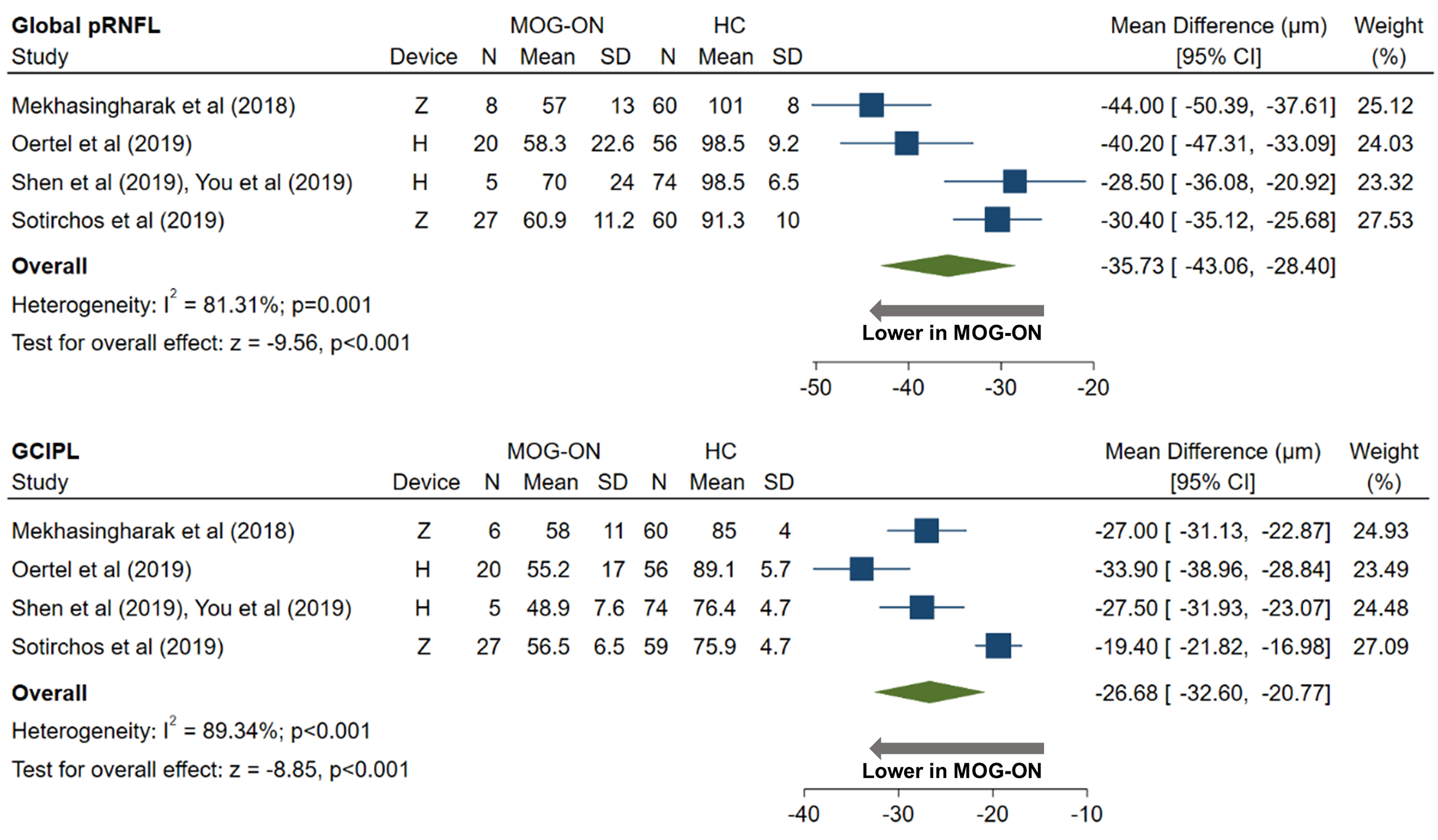

Supplement: Supplementary Figure 4 — Forest plot of the mean difference in global pRNFL and GCIPL thickness between MOG-ON and HC. [file Image_4.TIF]

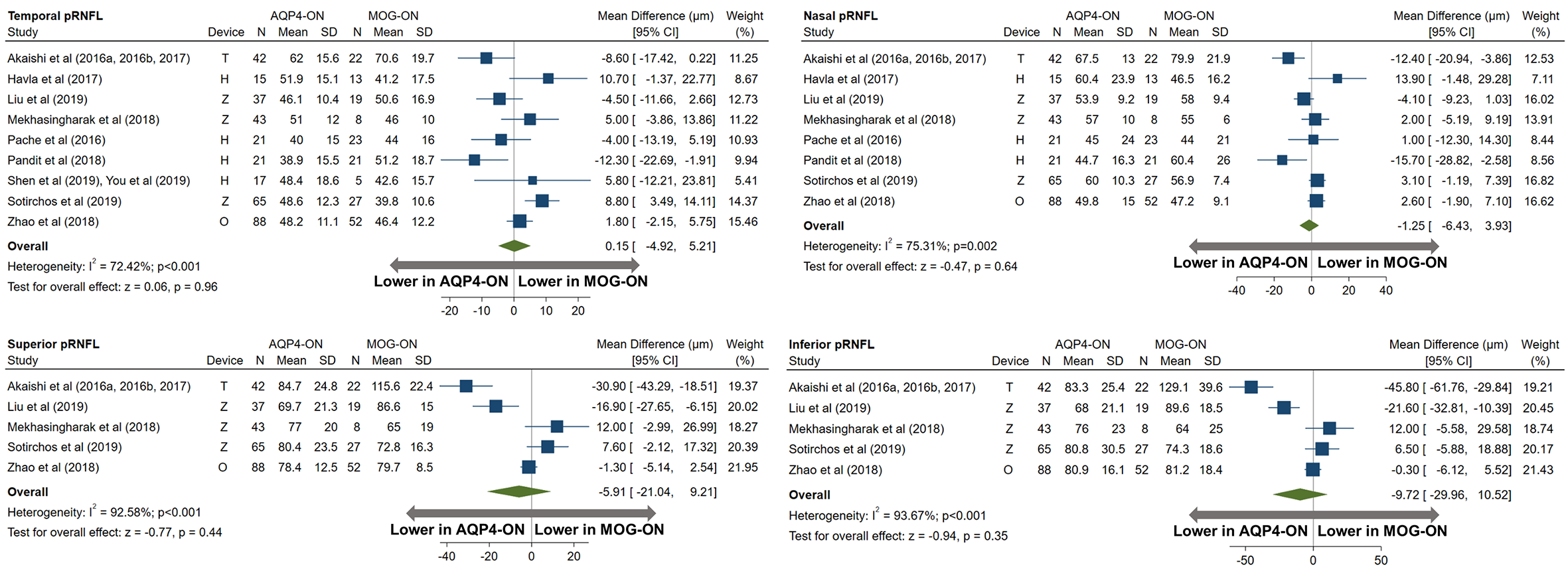

Supplement: Supplementary Figure 5 — Forest plot of the mean difference in quadrantal pRNFL thicknesses between AQP4-ON and MOG-ON. [file Image_5.TIF]

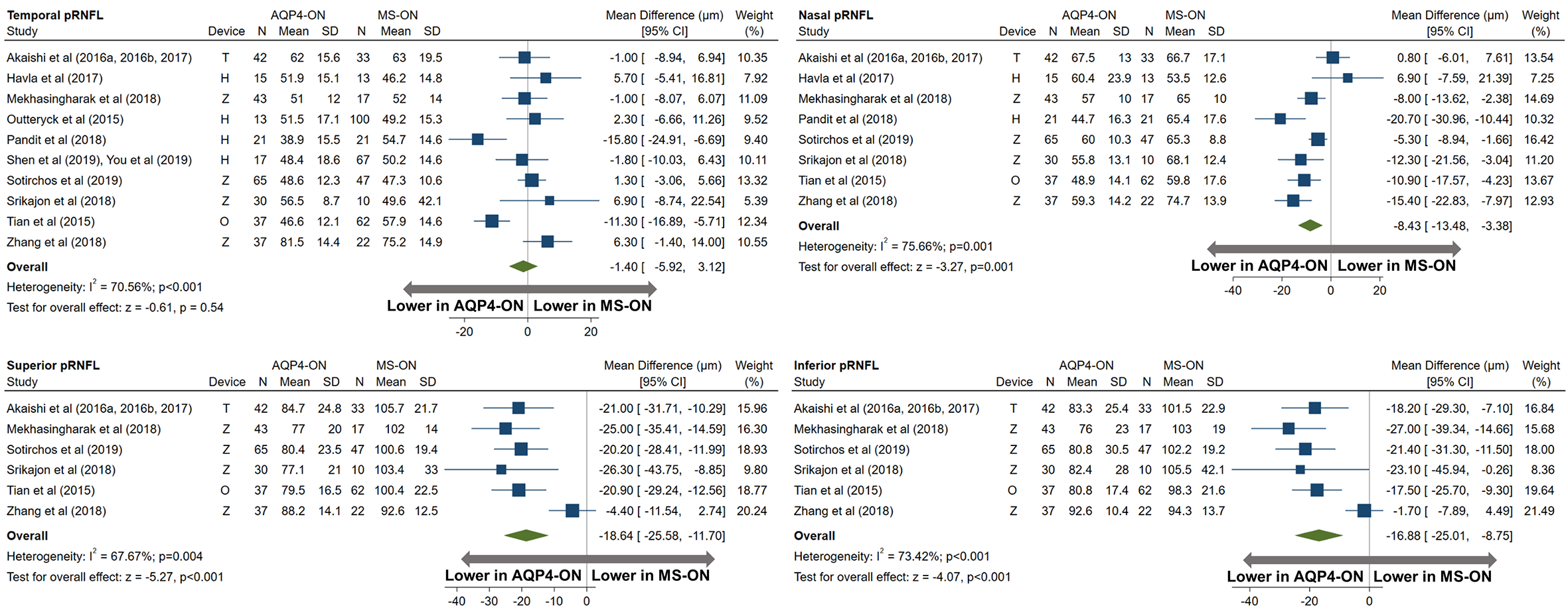

Supplement: Supplementary Figure 6 — Forest plot of the mean difference in quadrantal pRNFL thicknesses between AQP4-ON and MS-ON. [file Image_6.TIF]

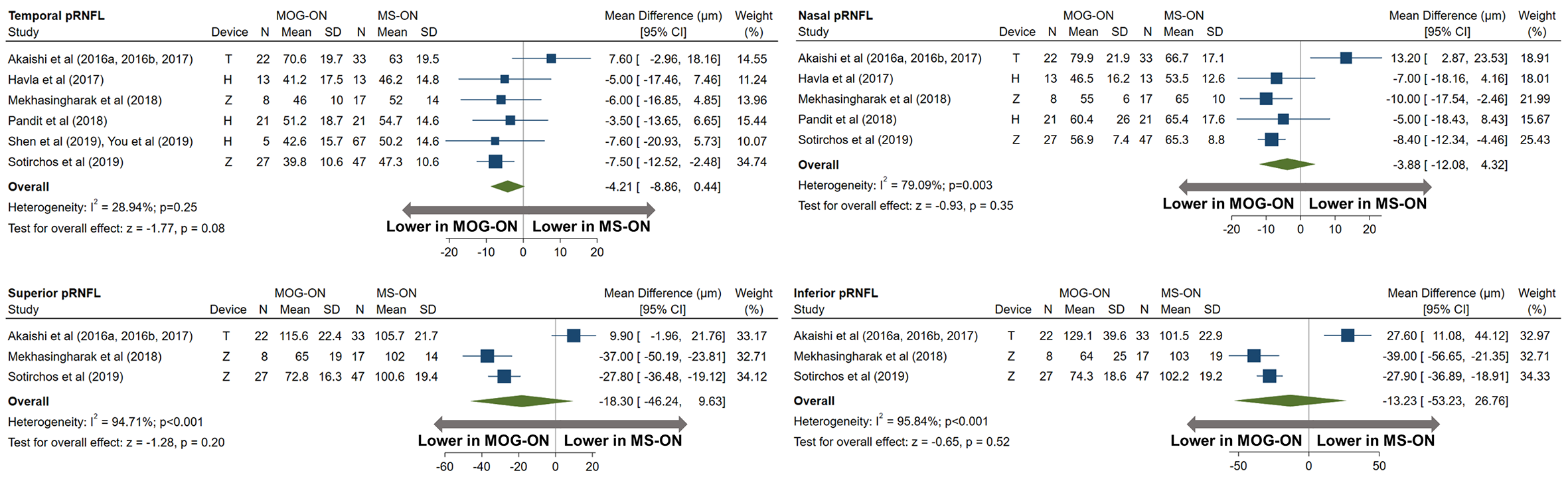

Supplement: Supplementary Figure 7 — Forest plot of the mean difference in quadrantal pRNFL thicknesses between MOG-ON and MS-ON. [file Image_7.TIF]

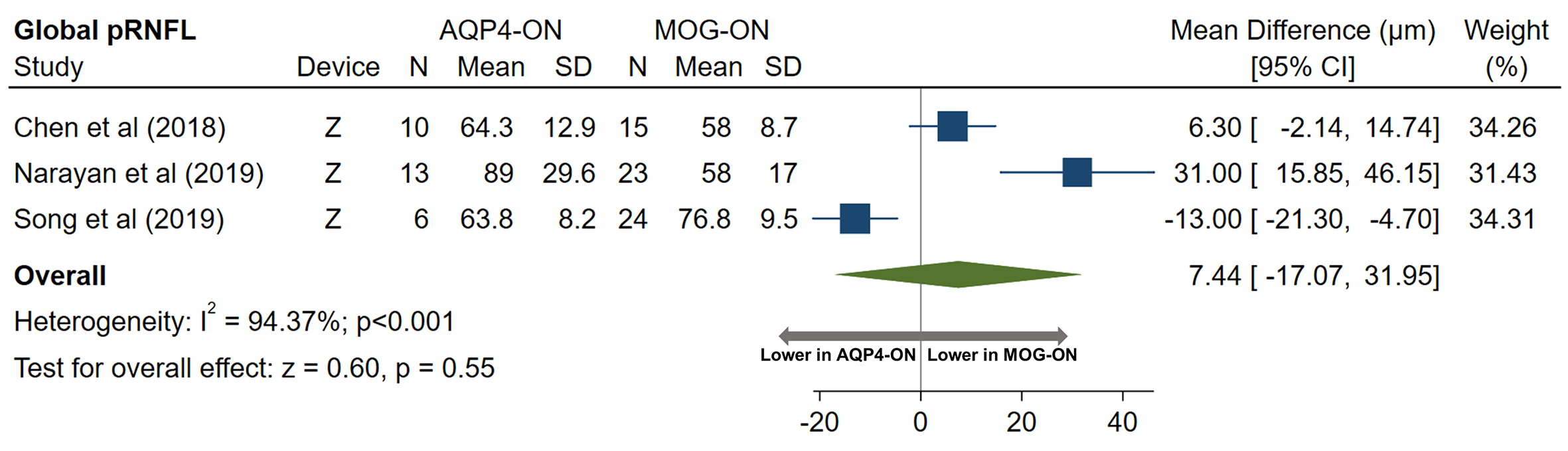

Supplement: Supplementary Figure 8 — Forest plot of the mean difference in global pRNFL thickness between AQP4-ON and MOG-ON in pediatric ON. [file Image_8.TIF]

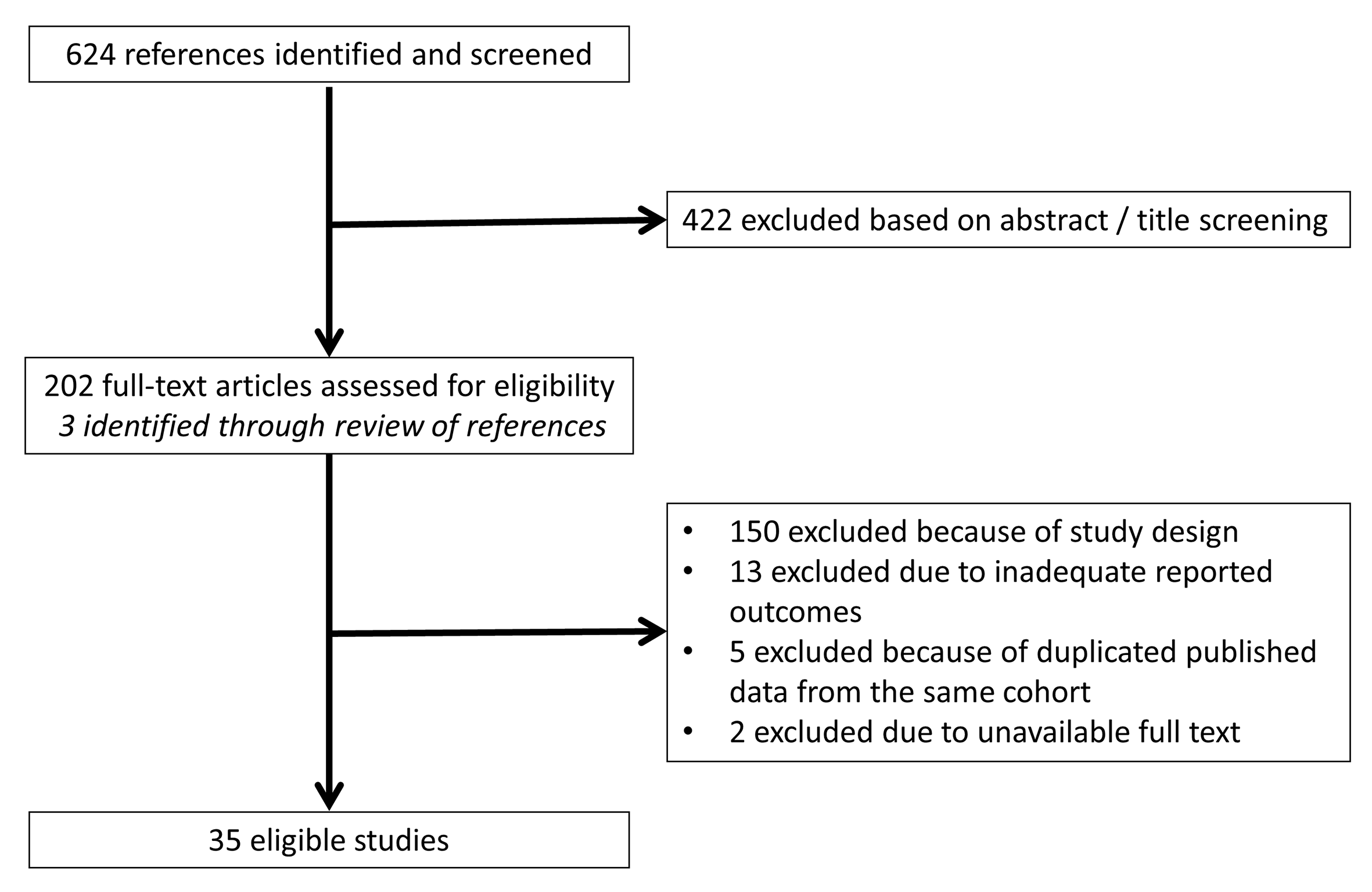

Supplement: Supplementary Figure 9 — Study selection for our third study objective (assessment of the visual outcome in AQP4-ON, MOG-ON and MS-ON eyes). [file Image_9.TIF]

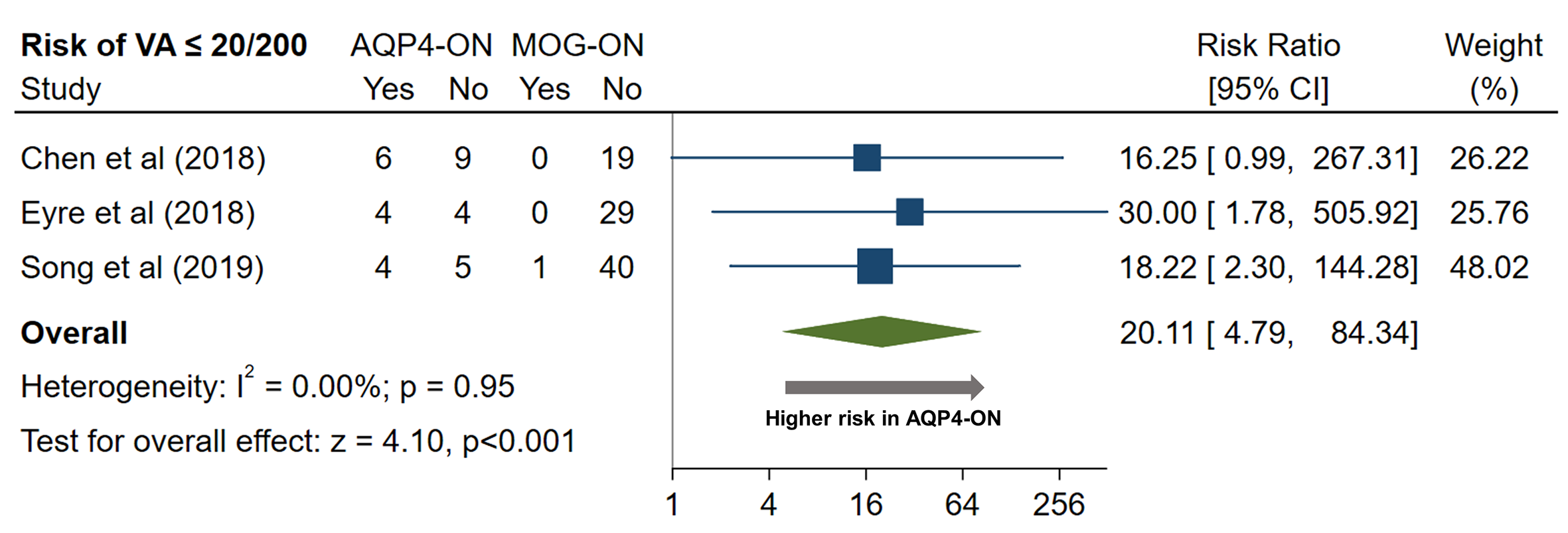

Supplement: Supplementary Figure 10 — Forest plot of the relative risk of a poor visual outcome (VA worse than 20/200) in AQP4-ON vs MOG-ON in pediatric ON. [file Image_10.TIF]
