# Supplementary material for: AQP4-IgG and MOG-IgG Related Optic Neuritis—Prevalence, Optical Coherence Tomography Findings, and Visual Outcomes: A Systematic Review and Meta-Analysis
Source: Front Neurol. 2020 Oct 8;11:540156. doi: 10.3389/fneur.2020.540156 (PMC7578376; doi:10.3389/fneur.2020.540156)
Supplement: Supplementary Table 1 — Search terms. [file Table_1.docx]

| **Study objective** | **Search term** |
| --- | --- |
| Assessment of the prevalence of AQP4-IgG and MOG-IgG seropositivity in isolated ON | ("mog" or "myelin oligodendrocyte glycoprotein" or "nmo" or "neuromyelitis optica" or “aquaporin 4” or “aqp4” or “aquaporin-4”) AND ("optic neuritis") |
| Comparison of OCT measures between AQP4-ON, MOG-ON and MS-ON eyes | ("mog" or "myelin oligodendrocyte glycoprotein" or "nmo" or "neuromyelitis optica" or “aquaporin 4” or “aqp4” or “aquaporin-4”) AND ("optical coherence tomography" or “retina” or “nerve fiber layer” or “ganglion cell”) |
| Assessment of the visual outcome in AQP4-ON, MOG-ON and MS-ON eyes | ("mog" or "myelin oligodendrocyte glycoprotein" or "nmo" or "neuromyelitis optica" or “aquaporin 4” or “aqp4” or “aquaporin-4”) AND ("optic neuritis") AND ("vision" or "visual" or "outcome" or "disability") |
