# Supplementary material for: AQP4-IgG and MOG-IgG Related Optic Neuritis—Prevalence, Optical Coherence Tomography Findings, and Visual Outcomes: A Systematic Review and Meta-Analysis
Source: Front Neurol. 2020 Oct 8;11:540156. doi: 10.3389/fneur.2020.540156 (PMC7578376; doi:10.3389/fneur.2020.540156)
Supplement: Supplementary Table 2 — Visual outcome in AQP4-ON, MOG-ON and MS-ON; qualitative synthesis. [file Table_2.docx]

eTable 2. Visual outcome in AQP4-ON, MOG-ON and MS-ON; qualitative synthesis

| **Study** | **Visual outcome scale** | **Outcome in AQP4-ON** | **Outcome in MOG-ON** | **Outcome in MS-ON** |
| --- | --- | --- | --- | --- |
| Cobo-Calbo et al (2018) | Visual acuity ≤ 0.2 | 29/66 patients (44%) | 11/136 patients (8%) | - |
| Carnero Contentti et al (2019) | Counting fingers (CF) or worse | 3/17 patients (18%) | - | 0/14 patients (0%) |
| Ishikawa et al (2019) | Counting fingers (CF) or worse | 12/54 patients (22%) | 2/39 patients (5%) | - |
|  | Visual acuity ≤ 0.5 | 24/54 patients (44%) | 7/39 patients (18%) | - |
|  | Visual acuity ≤ 0.2 | 30/54 patients (56%) | 10/39 patients (26%) |  |
| Kim et al (2019) | Mean logMAR | 0.75 | - | 0.10 |
| Kitley et al (2014) | Visual acuity ≤ 0.08 | 2/6 patients (33%) | 0/4 patients (0%) | - |
| Petzold et al (2019) | Decimal acuity [median (IQR)] | - | 0.58 (0.10-0.67) | 0.67 (0.33-1.00) |
| Piccolo et al (2016) | VFSS [median (range)] | 4 (0-5) | 0 (0-6) | 0 (0-5) |
| Ramanathan et al (2016) | VFSS of 6 | 3/11 patients | 0/19 patients | 0/13 patients |
|  | Final VFSS [mean; median (range)] | 2.2; 1 (0-6) | 1.4; 1 (0-5) | 0.6; 0 (0-2) |
| Sepúlveda et al (2016) | Visual acuity ≤ 0.2 | 41/94 patients (44%) | 1/9 patients (11%) | - |

CF: Counting fingers; VFSS: visual function system score; logMAR: logarithm of the minimum angle of resolution; IQR: interquartile range; AQP4: aquaporin 4; MS: multiple sclerosis; MOG: myelin oligodendrocyte glycoprotein; ON: optic neuritis
